# Finite element analysis-based flatness control and bending force optimization strategy for high rolling force conditions

**DOI:** 10.1371/journal.pone.0356630

**Published:** 2026-09-11

**Authors:** Zhuwen Yan, Wenjun Cao, Baosheng Wang, Yingxin Tang, Jiawei Wu, Shuyi Zhang

**Affiliations:** School of Mechanical Engineering, Nanjing Institute of Technology, Nanjing, Jiangsu, China; TU Dublin Blanchardstown Campus: Technological University Dublin - Blanchardstown Campus, IRELAND

## Abstract

To address severe flatness control failure of cold rolled strips under ultra-high rolling force, a collaborative bending force multi-objective optimization strategy based on surrogate models is proposed for a 1450 six-high tandem cold mill. Latin hypercube sampling (LHS) is adopted to generate process sample points, and Gaussian process regression (GPR) and radial basis function neural network (RBFNN) are constructed as fast surrogate models to replace time-consuming ABAQUS finite element simulation. NSGA-II and particle swarm optimization (PSO) are combined to perform global constrained optimization of work roll bending (WRB) and intermediate roll bending (IRB) forces, aiming to minimize transverse thickness deviation, quadratic/quartic strip crown, flatness residual error and rolling force unevenness simultaneously. Finite element verification shows that under high rolling force of 25000 kN, the optimized collaborative bending force combination reduces strip transverse thickness difference from 18 μm to 6 μm, quadratic crown from +11 μm to −5 μm, quartic crown from 3 μm to 1 μm, rolling pressure unevenness by 75.9%, and almost eliminates edge wave flatness defects. Mechanism analysis reveals that high rolling force significantly shifts the optimal single bending force operating window and increases quartic crown proportion, while intermediate roll bending force presents stronger anti-interference performance for quarter-wave defects. The proposed surrogate model-based optimization framework effectively overcomes the compensation limit of single bending force control under extreme heavy load, providing theoretical and technical support for high-precision flatness control of ultra-thin high-strength cold rolled strips.

## 1. Introduction

### 1.1 Background and significance of the study

The rapid development of industries such as high-end equipment manufacturing, new energy vehicles, and precision electronics has raised unprecedented high standards for cold-rolled strip steel, which serves as a crucial basic material. Its core quality indicators, namely plate shape and thickness accuracy, directly determine the success or failure of subsequent processes such as stamping and welding, as well as the performance of the final product. In order to meet the growing market demand for high-strength, ultra-thin strip steel, modern cold tandem rolling technology is continuously evolving towards higher speeds and greater precision.

Studying the formation mechanism, evolution law, and high-precision control strategy of strip shape under extreme rolling conditions is not only an important supplement and improvement to rolling theory, but also an urgent engineering demand to break the bottleneck of high-end strip steel production and enhance the core competitiveness of China’s advanced manufacturing industry. This project takes a 1450 six-high cold tandem mill as the research object, focusing on the core characteristic of large rolling force, aiming to reveal its strip shape regulation mechanism, and provide theoretical basis and technical solutions for achieving high-precision strip flatness control under extreme conditions. It has important theoretical significance and great engineering application value.

### 1.2 Research status at home and abroad

Researchers worldwide have conducted decades of in‑depth studies on strip‑shape detection [1,2], theoretical modeling [3,4], and control strategies [5,6], achieving substantial progress.

Domestic research started relatively late but has developed rapidly. Universities such as Northeastern University, the University of Science and Technology Beijing, Yanshan University, and enterprises such as Baowu and Ansteel have carried out extensive work. The ultra‑thin strip research group at Northeastern University [7–9] has systematically studied rolling methods for ultra‑thin strip and rolling equipment, proposing a novel concept of combined forming: under the joint influence of pressure reduction, asynchronous rolling, and front‑rear tension [10], a pressure‑shear‑tension combined forming condition is created [11]. Song Meng and others [12] developed a reversible asynchronous rolling process using a self‑designed four‑high mill, achieving ultra‑thin strip production without intermediate annealing and breaking the traditional minimum achievable thickness limit. Sims [13] derived a simplified rolling‑force formula based on rolling plastic deformation conditions. Oh and Kobayashi [14,15] developed a graphical method and, based on the extremum principle for rigid‑perfectly‑plastic materials, studied optimal numerical solutions for rolling force.

Swiss engineer Mathey [16–18] invented the first small six‑high mill in the early 20th century. Its symmetric support‑roll arrangement effectively limited work‑roll bending, but the structure had a critical drawback: when the work‑roll diameter was reduced to a certain level, the outer surface of the roll contacted the rolling line, restricting the usable roll‑diameter range. Building on the six‑high mill, German scholar Rohn [19–21] designed the world’s first twelve‑high mill by increasing the number of rolls to control strip shape and thickness, greatly improving the rolling efficiency of ultra‑thin strip. However, due to the lack of multi‑support‑beam structure, the overall rigidity of the twelve‑high mill was insufficient, making it unsuitable for wider ultra‑thin strip production. Che Jun and others [22–24] simplified the rolling deformation zone based on the Orowan theory and derived a rolling‑force model for composite strip, verifying via ABAQUS finite‑element simulation that the model’s error could be kept within 10%. Zhang Hongji and colleagues [25] established a finite‑element model for powder rolling using MSC.Marc, achieving simulation errors within 9.49%.

Internationally, Sajan Kapil and others [26] used finite element methods to establish work‑roll motion equations for a four‑high mill and analyzed vibrations induced by different process parameters. Jeon and others [27,28] studied deformation characteristics of Al‑Cu clad sheet rolling using physical modeling and FEM, proposing an analytical model for clad rolling. Zhao Qiang [29–31] established velocity and strain‑rate fields for large cylindrical shell rolling based on the upper‑bound method, achieving rolling‑force prediction accuracy within 10%. Gao Huimin [32–35] proposed a hybrid rolling‑force prediction method combining FEM and neural networks, where FEM generates training data for the neural network, achieving prediction errors within 10%.

## 2. Shape theoretical basis and defect formation mechanism in extreme rolling processes

Extreme rolling process refers to the strip steel rolling conducted under harsh process parameters such as high-strength materials, extremely large rolling force, extremely wide plate width, and extremely high speed. Such processes break the equilibrium assumption of traditional rolling theory, making plate flatness control the core difficulty. This chapter will systematically elaborate on its theoretical basis and the causes of defects.

### 2.1 Shape definition

Strip shape is one of the core indicators for measuring the quality of cold-rolled strip steel, which comprehensively reflects the lateral thickness distribution of the strip steel in the width direction and its flatness performance in downstream processes.

Horizontal Thickness Distribution: Horizontal thickness distribution refers to the thickness values of each point on the cross-section of strip steel perpendicular to the rolling direction. The ideal horizontal thickness distribution is a straight line parallel to the reference line, indicating that the thickness of each point on the strip steel is consistent with the target thickness. However, in the actual rolling process, due to factors such as elastic deformation, wear, and thermal expansion of the rolls, the actual shape of the roll gap is not ideal and straight, resulting in different reduction rates at the edges, middle, and quarter points of the strip steel, thus generating thickness deviations. This deviation is usually quantified by the following indicators: Wedge Shape: Refers to the thickness difference between the operating side and the drive side of the strip steel, which is an indicator to measure rolling asymmetry. Crown: Usually refers to the difference between the thickness at the midpoint of the width and the average thickness of the marked points on both sides.


$$\begin{array}{c} CR=h_{c}-\frac{h_{edl}+h_{eol}}{\text{2}} \end{array}$$

Where: CR: strip crown; $h_{edl}$: thickness at the drive side mark point; $h_{eol}$: thickness at the operator side mark point; $h_{c}$: thickness at the center point in the width direction.

Flatness: Flatness refers to the characteristic of whether the rolled strip steel is flat in a free state. When the longitudinal extension of each point on the strip steel is not consistent in the transverse direction, residual internal stress will be generated inside the strip steel. If this internal stress exceeds the critical buckling stress of the strip steel, the strip steel will become unstable and produce visible waves. Flatness usually refers to the relative length difference between the wavy part and the flat part of the plate and strip, multiplied by an amplification factor:


$$\begin{array}{c} I=\frac{\Delta L}{L}\times 10^{\text{5}} \end{array}$$

Where, ΔL is the length difference between the wavy part and the flat part, L is the length of the flat part. 10^5^ is a fixed amplification factor to make the result a convenient value for reading and comparison.

**Fig 1:**
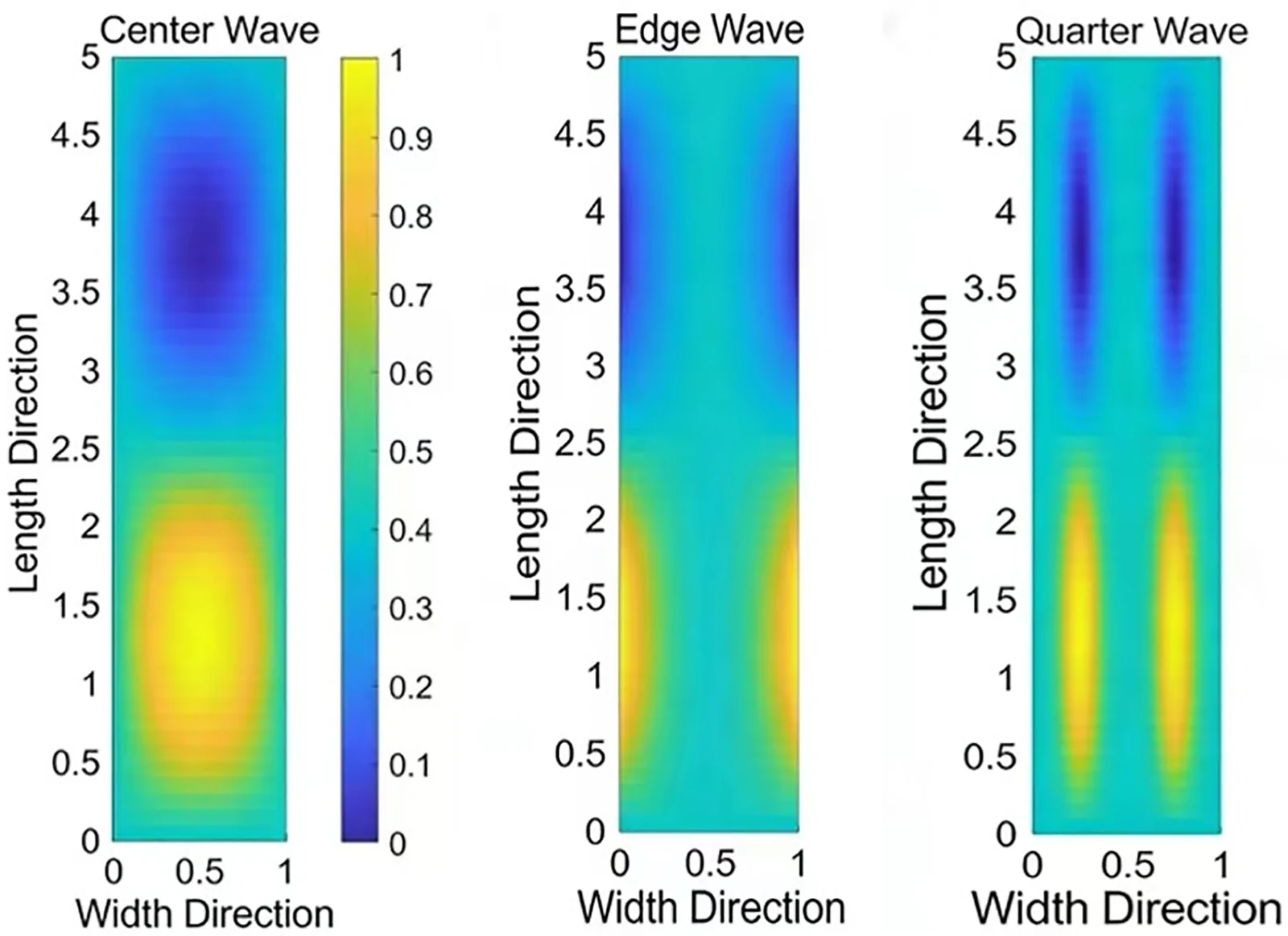
Strip shape defects.

Common plate shape defects include: edge wave: the edge extension is greater than the middle part, usually caused by excessive rolling force, excessive positive roll bending of the work roll, or insufficient roll crown. Middle wave: the middle extension is greater than the edge, usually related to excessive negative roll bending of the work roll or excessive thermal roll crown. Quarter wave: the quarter extension is too long, which is a typical defect in six-high mills, closely related to the position of the intermediate roll and the control of roll bending force. The flatness defects are shown in Fig 1.

The ultimate goal of flatness control is to achieve the highest possible flatness while ensuring the target crown, i.e., to ensure equal elongation rates at all transverse points of the strip.

### 2.2 Roll system deformation principles under high rolling force

In a six-high mill, extreme rolling forces are transmitted through the path of strip steel – work roll – intermediate roll – backup roll, inducing complex elastic deformation of the entire roll system. This deformation is the fundamental reason for the deviation of the roll gap shape from the ideal state, thereby affecting the plate shape.

According to Hertz’s contact theory, the elastic flattening of the rollers occurs when two rollers in contact and under pressure undergo significant elastic flattening deformation within the contact area. The width of this deformation zone can be estimated using Hertz’s formula:


$$\begin{array}{c} b=\sqrt{\frac{2F}{\pi L}\cdot \frac{\frac{1-\upsilon_{\text{1}}^{\text{2}}}{E_{\text{1}}}+\frac{1-\upsilon_{\text{2}}^{\text{2}}}{E_{\text{2}}}}{\frac{\text{1}}{D_{\text{1}}}+\frac{\text{1}}{D_{\text{2}}}}} \end{array}$$

Where, F is the rolling force, L is the barrel length, and E, D are the Young’s modulus, Poisson’s ratio, and diameter of the roll, respectively. The greater the rolling force and the smaller the roll diameter, the more severe the flattening. The work roll has the smallest diameter, so its flattening effect is most significant, directly altering the actual contact arc length and pressure distribution between the work roll and the strip, making it a key factor affecting the thickness uniformity of the rolled strip.

Under the bending moment generated by the rolling force, all rollers undergo bending deformation similar to that of a simply supported beam. Among them, the slender work rollers and intermediate rollers exhibit the most pronounced bending. This bending leads to different roll gap values between the middle and edge parts of the roller body, which is the main cause of strip steel crown or concavity. The maximum deflection can be approximately estimated using the beam bending formula in material mechanics:


$$\begin{array}{c} \delta_{max}\approx \frac{5qL^{\text{4}}}{384EI} \end{array}$$

Where, q is the distributed load of rolling force along the barrel length, E is the roll elastic modulus, and I is the cross-sectional moment of inertia of the roll. Clearly, rolling force F is the dominant factor for deflection. Under extreme rolling force, even in six-high mills with powerful backup roll support, the bending deflection of work rolls and intermediate rolls cannot be ignored and must be compensated by the bending roll system.

The final actual roll gap shape is the result of the combined superposition of the original roll crown, thermal crown, flattening deformation, and bending deformation. Extreme rolling forces greatly amplify the flattening and bending effects, leading to a decrease in the “stiffness” of the roll gap, that is, an increase in the amount of roll gap change caused by a unit change in rolling force. This makes the roll gap more sensitive to internal and external disturbances, exponentially increasing the difficulty of flatness control. An accurate roll system deformation model is the foundation for analyzing shape issues, which is also the necessity for the subsequent finite element simulation using ABAQUS.

### 2.3 Dominant factors for shape defect generation under extreme rolling conditions

In conventional rolling processes, plate shape defects can be effectively suppressed through measures such as roll bending and roll tilting. However, under extreme rolling force conditions, the following factors become the dominant contributors to plate shape defects, and they exhibit a strong coupling effect:

The distribution of rolling force is extremely uneven: extreme rolling force exacerbates the bending and flattening of the roll, but this deformation is not uniform across the length of the roll body. At the edges of the strip steel, due to the “edge effect,” the rolling force suddenly decreases, resulting in significant differences in the amount of roll flattening and bending deflection in this area compared to the middle part. This lateral uneven distribution of rolling force directly translates into uneven lateral extension of the strip steel, which is the most direct cause of severe edge waves or compound wave patterns.

The control efficacy of conventional bending force diminishes: The bending system artificially alters the deflection shape of the roll by applying hydraulic pressure at both ends of the roll, which is the core means of dynamically compensating for rolling force disturbances. However, under extreme rolling forces, the bending stiffness of the roll is relatively “softened.” To correct the bending caused by the huge rolling force, the required bending force must also be very large, and may even approach the upper limit of the hydraulic system’s capacity. At this point, the efficacy of the bending force decreases, the control sensitivity diminishes, and phenomena such as “inability to adjust” or “overshoot” may occur.

Drastic increase in contact pressure between rollers and conversion of frictional energy: The enormous rolling force leads to a drastic increase in contact pressure between the work roller and the intermediate roller, as well as between the intermediate roller and the backup roller. This not only exacerbates the wear of the rollers, but more importantly, the huge frictional work generated under high contact pressure is converted into heat energy, causing a sharp change in the local thermal crown of the rollers. The change in thermal crown will be superimposed on the mechanical deformation, further distorting the shape of the roll gap. This thermal effect exhibits time lag and nonlinearity, making flatness control more complex and difficult.

Changes in the plastic deformation behavior of strip steel: For the materials specified in the specifications, under extreme rolling forces, the deformation resistance, work hardening effect, and deformation permeability of the strip steel will all undergo changes. The differences in stress-strain states at various points on the cross-section of the strip steel are amplified, making it more prone to uneven flow at the microscale, which manifests macroscopically as stubborn shape defects that are difficult to eliminate through conventional means.

## 3. Finite element model establishment and shape analysis

### 3.1 ABAQUS finite element model establishment

**Fig 2:**
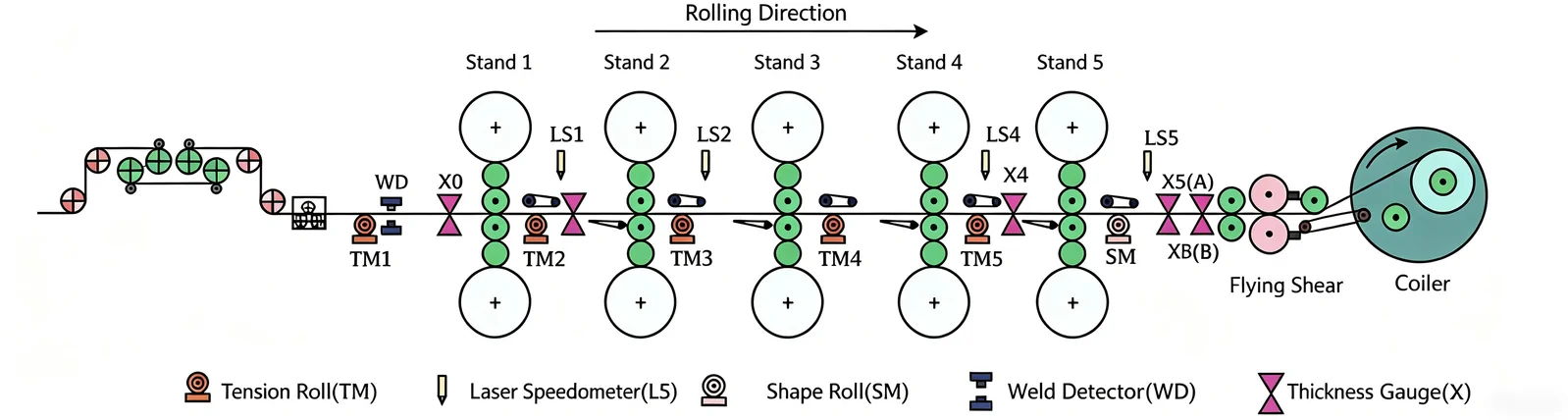
Six-high tandem cold mill model.

To achieve high-precision simulation of extreme rolling processes, it is necessary to establish a finite element model that accurately reflects the complex roll system structure, contact relationships, and material nonlinear behavior. Solid models of the backup roll, intermediate roll, and work roll were established separately. Table 1 lists the parameters of each component of the six-high mill. The overall equipment layout is shown in Fig 2.

**Table 1:** Cold rolling model parameters.

Parameter Name	Data
**Strip Entry Thickness at Stand 1**	4 mm
**Strip Exit Thickness at Stand 5**	2 mm
**Work Roll Barrel Diameter**	480 mm
**Work Roll Neck Diameter**	360 mm
**Work Roll Barrel Length**	1450 mm
**Intermediate Roll Barrel Diameter**	600 mm
**Intermediate Roll Neck Diameter**	500 mm
**Intermediate Roll Barrel Length**	1450 mm
**Backup Roll Barrel Diameter**	1300 mm
**Backup Roll Neck Diameter**	720 mm
**Backup Roll Barrel Length**	1450 mm
**Strip Length**	1000 mm
**Friction Coefficient on All Rolling Surfaces**	0.1
**Initial Strip Speed**	1100 mm/s

The figure clearly illustrates the three-tier roller system structure (work roll + intermediate roll + backup roll) of the six-high mill. During simulation modeling, it is necessary to establish solid models for each of the three types of rolls, restoring the contact relationships between rolls and between rolls and strip steel, in order to accurately simulate the elastic deformation of the roller system and the distribution of contact pressure during the rolling process. This provides a foundation for the deformation and force analysis of the strip steel. Equipment such as tension rolls, speedometers, and thickness gauges correspond to the closed-loop control logic for tension, speed, and thickness during the rolling process. In multi-field coupled simulations, these control logics can be embedded into the model through boundary conditions and load curves, restoring the actual rolling process constraints. Tension rolls: stabilize the tension between stands to avoid strip steel deviation and breakage, serving as the execution unit for tension closed-loop control in the continuous rolling process; laser speedometers: measure the running speed of strip steel in real time, providing core data for second flow rate constant control and speed cascade adjustment; thickness gauges: used for entry thickness reference, stand-to-stand thickness feedback, and finished product thickness closed-loop control, ensuring strip steel thickness accuracy; shape rolls at the exit of stand 5: detect strip steel shape defects, providing feedback signals for flatness control methods such as roll bending and roll shifting.

Since the rollers are set as elastic bodies, plastic deformation is not considered. The actual raw materials used by the factory are selected as the material properties for the simulated strip steel. It is determined that the density and elastic properties of the rollers and strip steel are the same, with a density of 7.85 g/cm3, a Young’s modulus of 160,000 MPa, and a Poisson’s ratio of 0.3.

In the field of finite element simulation, the quality of mesh generation has a decisive impact on computational accuracy and efficiency. Among them, first-order elements, as the basic mesh type, have nodes only distributed at the corners of the element, and rely on linear interpolation for computation. Each degree of freedom (DOF) direction is set with two integration points inside the element. Common first-order tetrahedral and hexahedral elements belong to this category. With their simple topological structure, these elements can achieve fast and efficient mesh generation, especially suitable for preliminary analysis of models or rapid modeling of large-scale structures. However, limited by their simple interpolation method and fewer node arrangements, they are prone to geometric description deviations and numerical calculation errors when dealing with complex deformations and stress distributions. Typical issues such as the frequent shear locking phenomenon in bending deformation scenarios may lead to an overestimation of structural stiffness, thereby affecting the accuracy and reliability of the computational results. Compared to first-order elements, second-order elements have nodes set at both ends and the midpoint of each edge, using quadratic interpolation for computation, with three integration points in each DOF direction, such as second-order tetrahedral and hexahedral elements. Their simulation effects are significantly better than those of first-order elements, allowing for better fitting of complex geometric shapes and precise capture of stress concentration. In the analysis of complex conditions such as thin-walled structures, they can more realistically reflect the mechanical properties of the structure.

**Fig 3:**
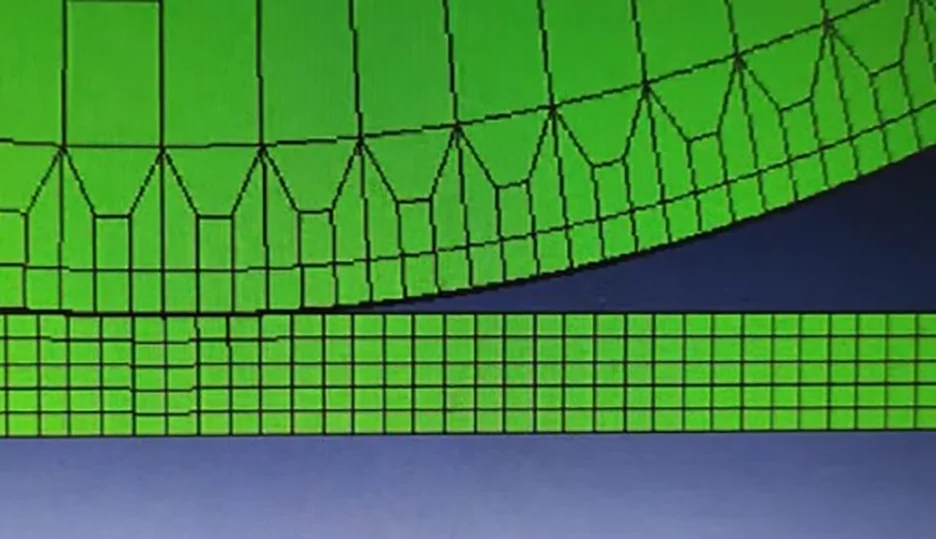
Three-inlet single-outlet mesh generation.

This article employs a second-order hexahedral element mesh. In the roll grid generation, a differentiated strategy is adopted for the work roll, intermediate roll, and backup roll: the middle part of the roll body section uses a coarse mesh, while the circumferential area is refined into a fine mesh. For the work roll, the mesh accuracy is further optimized. In the key areas in contact with the strip steel, a three-in-one grid generation method is employed, as shown in Fig 3, to achieve local densification. This division method significantly enhances the accuracy of strip steel rolling simulation and provides a reliable guarantee for in-depth analysis of the variation patterns during the rolling acceleration phase.

### 3.2 Analysis of rolling force influence on shape

As the most fundamental process parameter in the rolling process, the magnitude and distribution of rolling force directly determine the deformation state of the roll system and the strip shape quality of the steel strip. Based on elastic mechanics and rolling theory, this section systematically analyzes the mechanism of how rolling force affects various indicators of strip shape, providing a theoretical basis for subsequent optimization control.

In a six-high mill, the transmission of rolling force follows the path of “strip steel → work roll → intermediate roll → backup roll → stand.” According to the theory of elastic mechanics, the deformation of the roll system caused by rolling force mainly includes the following components:

Work Roll Elastic Bending Deformation: The maximum bending deflection of the work roll under rolling force can be expressed as:


$$\begin{array}{c} \delta_{WR}=\frac{P\cdot L^{\text{3}}}{48E_{WR}I_{WR}}+\frac{P}{K_{WR}} \end{array}$$

Where P is the total rolling force, L is the barrel length, $E_{WR}$ is the work roll elastic modulus, $I_{WR}$ is the work roll cross-sectional moment of inertia, and $K_{WR}$ is the work roll bending stiffness coefficient.

Inter-Roll Elastic Flattening Deformation: Based on Hertz contact theory, the elastic flattening between the work roll and intermediate roll is:


$$\begin{array}{c} \delta_{WI}=\frac{2P}{\pi b}\cdot \frac{1-v^{\text{2}}}{E}\cdot ln\left(\frac{4R_{WR}R_{IR}}{b^{\text{2}}}\right) \end{array}$$

Where b is the contact half-width, $R_{WR},R_{IR}$ are the work roll and intermediate roll radii, respectively,Eis the elastic modulus.

Backup Roll Deformation Characteristics: Due to their large diameter, backup rolls mainly exhibit overall bending deformation. Their influence on shape is indirect, affecting the boundary conditions of the intermediate and work rolls.

### 3.3 Processing and analysis of simulation results under initial settings

**Fig 4:**
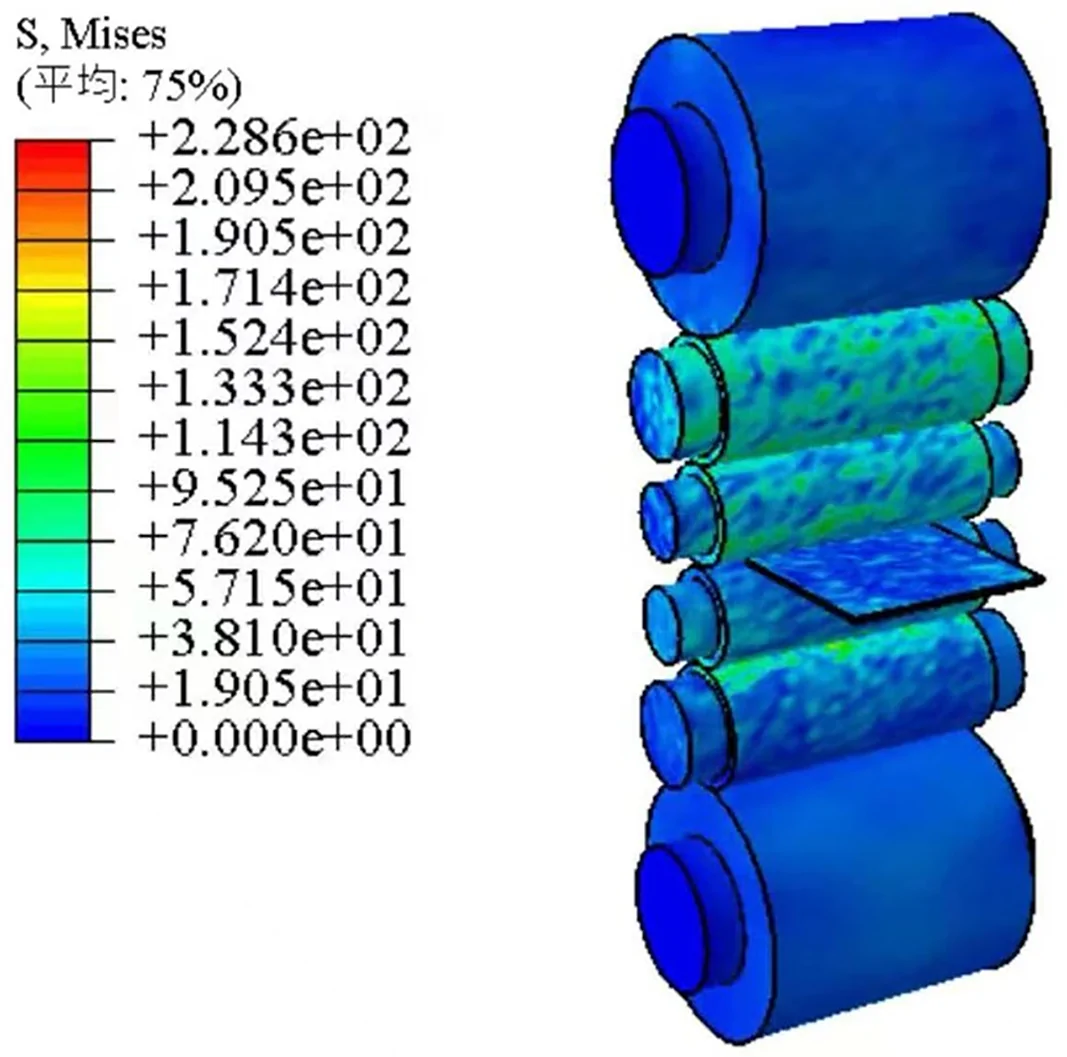
Stress diagram of the rolling mil.

**Fig 5:**
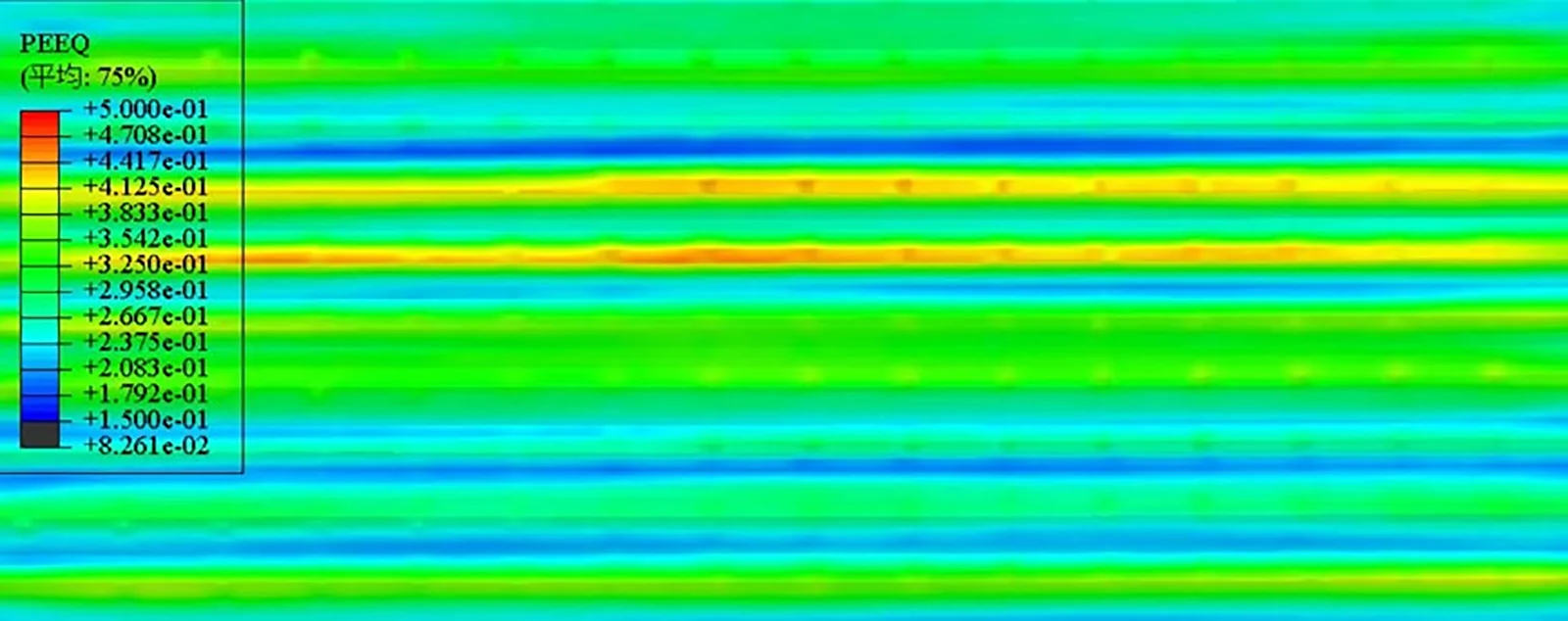
Strain contour plot.

Fig 4 depicts the force distribution across the entire rolling mill during the rolling process. The color bar ranges from 0 MPa to 2.286 × 10^2 MPa, with stress concentration occurring at the work roll neck. Fig 5 illustrates the plastic deformation of the rolled strip steel, with values ranging from 0.08 to 0.5. The colors transition from blue to red, indicating strain increasing from low to high. Most of the area is green and yellow, indicating moderate strain. Changes in rolling force due to variations in rolling time result in some alterations and fluctuations in the thickness and shape of the rolled strip steel.

## 4. Simulation results and analysis

Based on the ABAQUS finite element simulation platform, a systematic analysis was conducted on the influence of work roll bending force (WRB) and intermediate roll bending force (IRB) on strip shape quality under conventional and high rolling forces. By employing the method of controlled variables, the study investigated the response characteristics of strip steel outlet thickness distribution, crown, flatness, rolling force distribution, and inter-roll pressure when WRB varies at −100 KN, 0 KN, 100 KN, 200 KN, 300 KN, and 400 KN, and when IRB varies at 0 KN, 100 KN, 200 KN, 300 KN, 400 KN, and 500 KN.

### 4.1 Thickness influence analysis

The uniformity of strip steel’s transverse thickness distribution is a fundamental indicator of plate shape quality. This section reveals in detail the mechanism of the influence of bending force on thickness distribution through finite element simulation.

#### 4.1.1 Influence of work roll bending force on thickness under conventional rolling force.

Under conventional rolling force and fixed IRB, the transverse thickness distribution of the strip shows regular changes when WRB increases from −100 KN to 400 KN.

**Fig 6:**
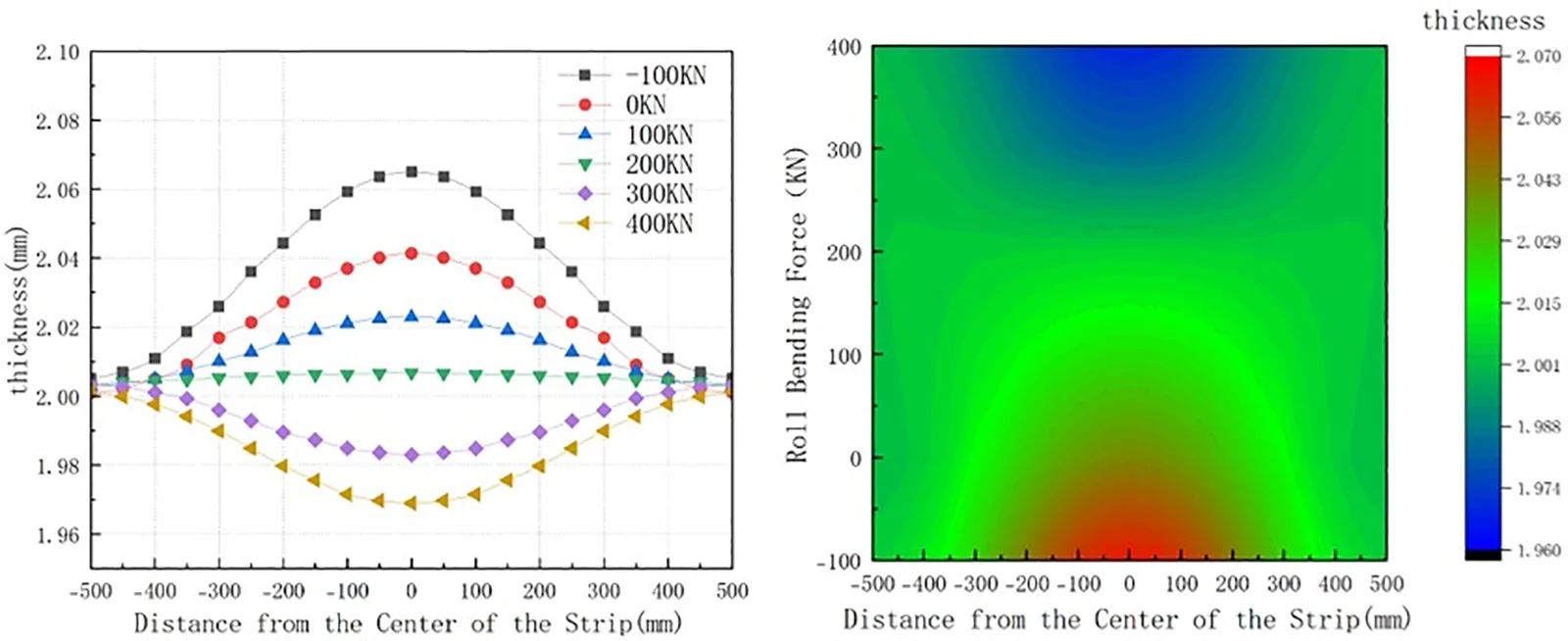
Effect of work roll bending force on strip thickness under conventional rolling force.

As can be seen from Fig 6, when WRB is −100 KN, the thickness distribution exhibits a distinct feature of being thicker at the edges and thinner at the center. The center thickness is 2.07 mm, while the edge thickness reaches 2.005 mm, with a thickness difference of 65 μm. This is due to the negative bending force exacerbating the bending deformation of the work roll, resulting in a convex roll gap. The negative bending force intensifies the bending deformation of the work roll, causing the roll gap to exhibit a convex shape. During the rolling process, the reduction in the central region of the strip steel is less than that at the edges, ultimately leading to a thickness distribution where the center is thicker and the edges are thinner. This convex thickness distribution directly induces edge wave defects, seriously affecting the shape quality of the strip steel. As WRB gradually increases, the thickness distribution curve tends to flatten. When WRB is 200 KN, the thickness distribution is most uniform, with a center thickness of 2.006 mm and an edge thickness of 2.003 mm, and a thickness difference of only 3 μm, achieving ideal flatness. The external moment generated by the positive bending force precisely offsets the bending deformation of the work roll caused by the rolling force, resulting in a flat roll gap shape and uniform reduction across the full width of the strip steel, laying the foundation for good shape quality. Continuing to increase WRB to 400 KN, the thickness distribution transitions to an inverted distribution where the center is thinner and the edges are thicker. The center thickness decreases to 1.97 mm, while the edge thickness increases to 2.001 mm, with a thickness difference of 31 μm. Excessively large positive bending force can overly offset the bending of the work roll caused by the rolling force, resulting in a concave roll gap. The reduction in the central region of the strip steel is much greater than that at the edges, ultimately leading to a concave thickness distribution where the center is thinner and the edges are thicker, which in turn induces mid-wave defects.

This variation pattern originates from the work roll bending mechanism: the external moment generated by the positive bending force can partially offset the work roll bending caused by the rolling force, making the roll gap shape tend to be straight; however, an excessive positive bending force can lead to overcompensation, resulting in a concave roll gap. Under conventional rolling forces, the optimal thickness uniformity is achieved when WRB = 200 KN, at which point the compensating effect of the bending force and the roll system deformation caused by the rolling force reach a balance.

The work roll bending force adjusts the shape of the roll gap by altering the bending moment distribution of the work roll. The external moment generated by positive bending force can partially offset the bending of the work roll caused by the rolling force, making the roll gap tend to be straight; negative bending force intensifies the bending of the work roll, leading to an increase in roll gap crown. The regular changes in color distribution in the contour plot visually reflect this adjustment process.

#### 4.1.2 Influence of work roll bending force on thickness under high rolling force.

When rolling force increases to 25000 KN, the influence of work roll bending force on thickness distribution significantly intensifies, and the optimal process window shifts.

**Fig 7:**
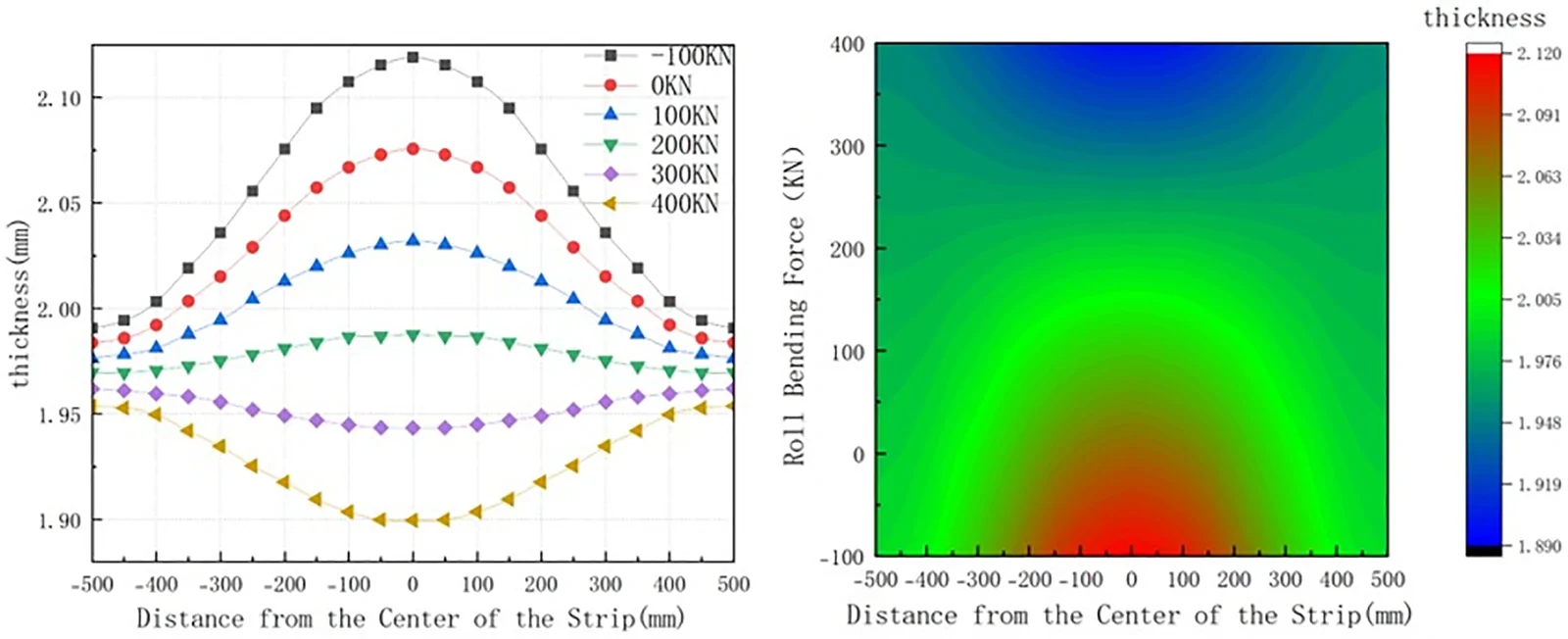
Effect of work roll bending force on strip thickness under high rolling force.

As shown in Fig 7, under high rolling force conditions, the thickness distribution fluctuations under all WRB settings are significantly greater than those under conventional conditions. When WRB is −100 KN, the center thickness reaches 2.12 mm, while the edge thickness is 1.99 mm, with a thickness difference of 130 μm, representing a 133% increase compared to conventional conditions. On the one hand, high rolling force itself significantly exacerbates the bending deformation of the work roll; on the other hand, negative bending force further amplifies this deformation, causing a sharp increase in roll gap crown, where the reduction in the central region of the strip steel is much smaller than that at the edges, ultimately resulting in an extremely convex thickness distribution, which is highly prone to causing severe edge wave defects. As WRB increases, the unevenness of thickness distribution gradually improves, but the rate of improvement is relatively slow. When WRB is 100 KN, the thickness distribution is relatively optimal, with a center thickness of 2.03 mm and an edge thickness of 1.97 mm, and a difference of 33 μm. It is worth noting that under high rolling force, the WRB value required to achieve optimal thickness uniformity shifts from the conventional value of 200 KN to 100 KN. Under high rolling force, the WRB value required to achieve optimal thickness uniformity shifts from 200 KN in conventional conditions to 100 KN, indicating a significant leftward shift in the process window. At this point, the compensatory effect of bending force and the roll system deformation induced by high rolling force reach a new equilibrium, and the thickness uniformity reaches its optimal level under these conditions, but it is still significantly inferior to the optimal state under conventional conditions.

This phenomenon can be explained from the perspective of roll system deformation mechanism: the large rolling force leads to a significant increase in the bending deformation of the work roll, and in order to maintain the flatness of the roll gap, a larger positive bending roll torque is required for compensation. However, the simulation results show that the optimal WRB (Work Roll Bending, work roll bending) actually decreases. The reason is that the large rolling force simultaneously exacerbates the flattening deformation between the rolls, altering the force balance point of the entire roll system. In addition, the absolute fluctuation of thickness distribution under high rolling force increases, indicating that relying solely on WRB is no longer sufficient to fully compensate for the roll system deformation caused by extreme loads. It is necessary to coordinate with other flatness control methods.

Under high rolling force, the WRB value corresponding to the optimal thickness shifts from the conventional 200 KN to 100 KN, reflecting the change in the equilibrium point of roll deformation caused by the increase in rolling force. The cloud chart comparison clearly demonstrates this shift characteristic. Even with the optimal WRB setting, the cloud chart still shows significant uneven thickness distribution under high rolling force, with yellow transitions in local areas indicating the presence of residual unevenness. This indicates that relying solely on work roll bending force is no longer sufficient to fully compensate for roll deformation caused by extreme loads.

#### 4.1.3 Influence of intermediate roll bending force on thickness under conventional rolling force.

Under conventional rolling force and fixed WRB, intermediate roll bending force (IRB) from 0 KN to 500 KN demonstrates strong control capability over thickness distribution.

**Fig 8:**
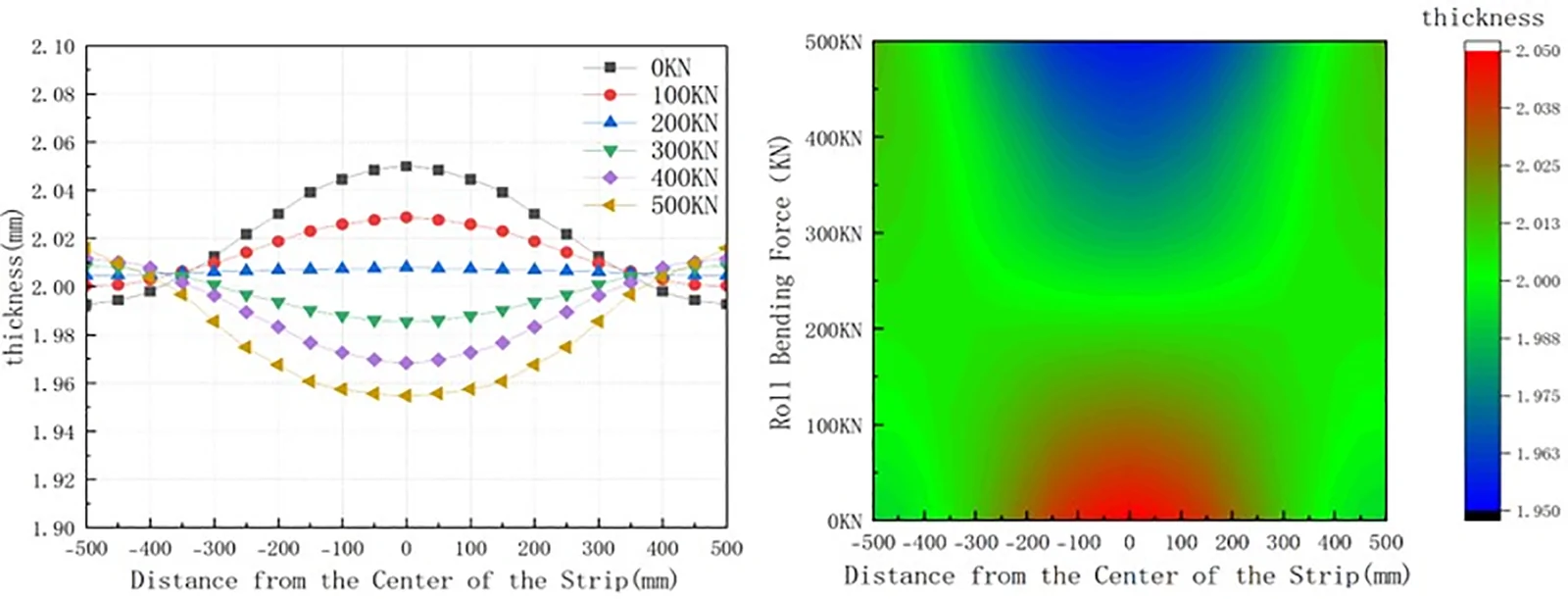
Effect of intermediate roll bending force on strip thickness under conventional rolling force.

As can be seen from Fig 8, when IRB = 0 KN, the thickness distribution exhibits a slight edge wave trend, with a center thickness of 2.05 mm and an edge thickness of 1.99 mm, resulting in a range of 60 μm. In this state, the bending deformation of the work roll and intermediate roll caused by the rolling force has not been effectively compensated, and there is a certain degree of convexity in the roll gap, leading to a smaller reduction in the central region of the strip steel compared to the edges, forming a mid-convex thickness distribution. As IRB increases, the thickness distribution curve undergoes significant changes. When IRB = 200 KN, the thickness distribution is most uniform, with a center thickness of 2.006 mm and an edge thickness of 2.003 mm, and a range of 3 μm. Continuing to increase IRB to 500 KN, the thickness distribution exhibits a clear center wave characteristic, with the center thickness decreasing to 1.95 mm and the edge thickness increasing to 2.01 mm, resulting in a range of up to 60 μm. Excessive bending force of the intermediate roll can overly offset the bending of the roll system caused by the rolling force, resulting in a concave roll gap, where the reduction in the central region of the strip steel is much greater than that in the edges, ultimately forming a mid-concave thickness distribution with reduced thickness in the center and increased thickness at the edges.

Compared to the work roll bending force, the intermediate roll bending force has a higher efficiency in regulating thickness distribution. For every 100 KN increase in IRB, the average central thickness changes by approximately 20 μm, while the same change in WRB only causes a central thickness change of about 1.5 μm. This is because the intermediate roll has a larger diameter and higher bending stiffness than the work roll, resulting in a more significant reverse bending effect when a bending force is applied. Additionally, the intermediate roll is positioned closer to the backup roll, allowing its bending deformation to be more effectively transmitted to the stand through the backup roll bearing pedestal, reducing energy dissipation.

This difference stems from the larger diameter and higher bending stiffness of the intermediate roll. The anti-bending effect is more pronounced when a bending force is applied. Furthermore, the position of the intermediate roll is closer to the support roll, allowing its bending deformation to be more effectively transmitted to the frame through the support roll bearing seat, reducing energy dissipation. The thickness control provided by the bending force of the intermediate roll is more “gentle” and “gradual,” with a smoother color transition in the contour plot, avoiding the sharp changes that may be caused by the bending force of the work roll. This provides a better foundation for fine flatness control.

#### 4.1.4 Influence of intermediate roll bending force on thickness under high rolling force.

Under high rolling force conditions, the control of thickness distribution by intermediate roll bending force faces greater challenges but also demonstrates its irreplaceable value.

**Fig 9:**
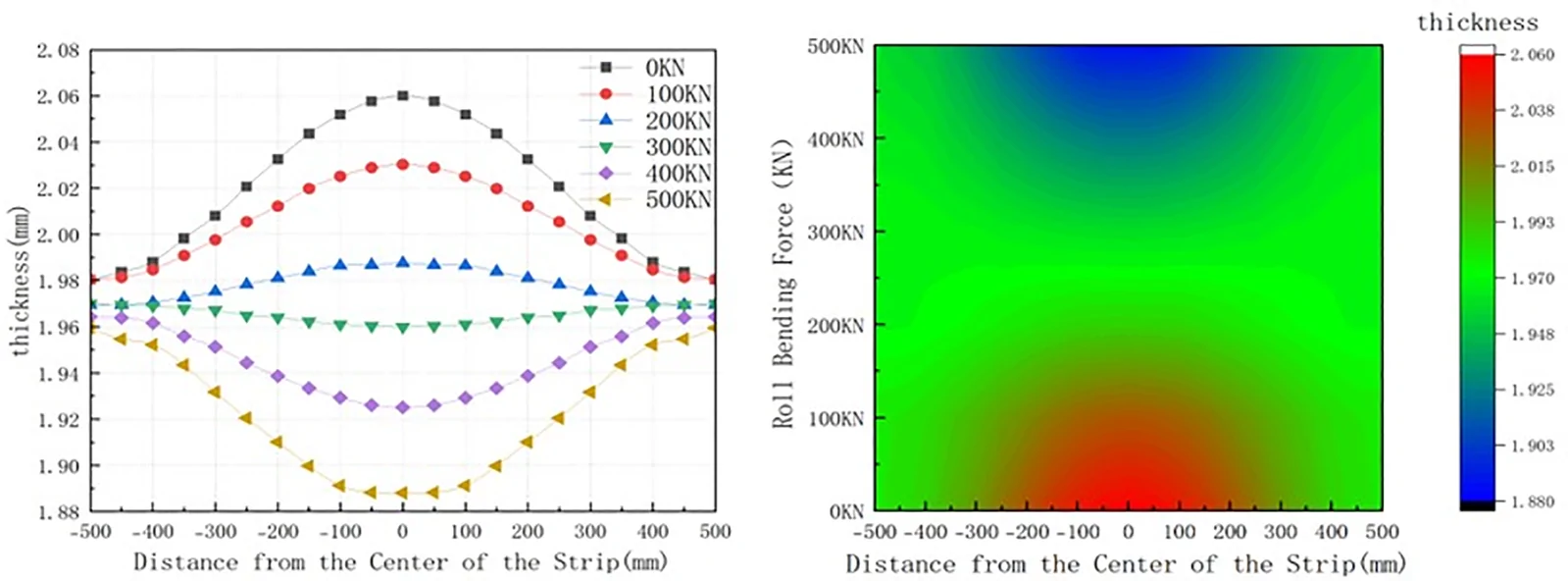
Effect of intermediate roll bending force on strip thickness under high rolling force.

As shown in Fig 9, the baseline level of thickness distribution undergoes significant changes under high rolling force. When IRB = 0 KN, the center thickness reaches 2.05 mm, the edge thickness reaches 1.99 mm, and the range is up to 60 μm, which is doubled compared to conventional conditions. Under heavy load conditions, the bending deformation of the work roll and intermediate roll caused by the rolling force is significantly enhanced. Without effective compensation torque under zero bending roll force, the roll gap crown increases sharply, and the reduction in the central area of the strip steel is much smaller than that at the edges, ultimately forming a strong mid-crown thickness distribution, posing a serious risk of edge wave defects. As IRB increases, the thickness distribution gradually improves, reaching a relatively optimal state when IRB = 200 KN, with a center thickness of 2.008 mm, an edge thickness of 2.004 mm, and a range of 4 μm. It is worth noting that even in this optimal state, the thickness distribution still fails to achieve complete flatness, and there is slight thickening at the edges. Excessive positive bending roll force can cause excessive compensation of the roll system bending, resulting in a concave roll gap and a sharp increase in inter-roll pressure, posing a serious threat to the lifespan of the rolling mill roll system and equipment. The appearance of high red value areas in the central region of the contour plot visually confirms the deteriorating trend of thickness distribution and clarifies the effective regulation upper limit of the intermediate roll bending force.

Compared to the work roll bending force, the intermediate roll bending force exhibits better anti-interference capability under high rolling force. When the IRB increases from 0 KN to 200 KN, the thickness variation decreases from 60 μm to 4 μm, with an improvement of 93%; whereas the improvement of WRB under the same conditions is only 70%. This indicates that under extreme rolling conditions, the intermediate roll bending force should be the preferred means for flatness control. However, there are limitations to the regulation of IRB. When the IRB exceeds 300 KN, the thickness distribution deteriorates, and the inter-roll pressure rises sharply, posing a threat to equipment life.

The regulation of the bending force of the intermediate roll exhibits notable limitations. When the Intermediate Roll Bending (IRB) exceeds 300 KN, the cloud diagram reveals the onset of red in the central region, indicating a deterioration in thickness distribution. Additionally, the pressure between the rolls escalates sharply, posing a threat to the lifespan of the equipment. Under high rolling forces, even with the optimal IRB setting, the cloud diagram still demonstrates residual unevenness. This underscores the necessity for a synergistic effect between the bending forces of the work roll and the intermediate roll to achieve optimal thickness uniformity.

### 4.2 Crown influence analysis

Strip steel crown is an important indicator for measuring the cross-sectional shape, directly affecting the quality of subsequent processing steps. In shape analysis, crown is usually decomposed into two components: second-order crown and fourth-order crown, each corresponding to different shape defect patterns. The second-order crown mainly reflects symmetrical edge waves or mid-waves, while the fourth-order crown reflects more complex quarter-wave patterns. This section quantitatively analyzes the regulation characteristics of roll-bending force on these two crown components through finite element simulation.

#### 4.2.1 Influence of work roll bending force on crown under conventional rolling force.

Under conventional rolling force and fixed IRB, work roll bending force from −100 KN to 400 KN shows different variation patterns for quadratic and quartic crown.

**Fig 10:**
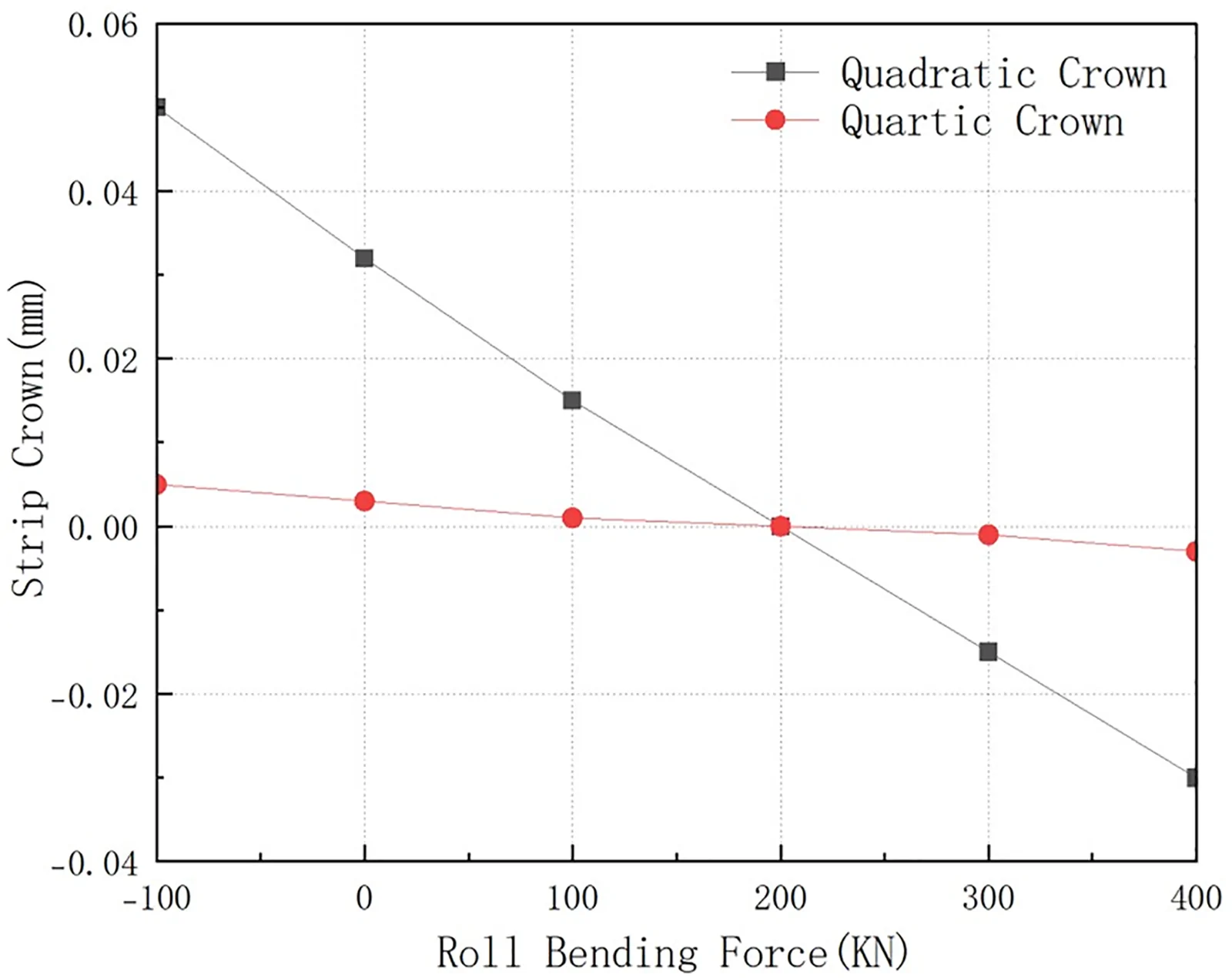
Effect of Work Roll Bending Force on Strip Crown Under Conventional Rolling Force.

As can be seen from Fig 10, under conventional rolling force, the work roll bending force has a significant effect on regulating the secondary crown, while its impact on the fourth-order crown is relatively limited. When the WRB (Work Roll Bending Force) increases from −100 KN to 400 KN, the secondary crown decreases linearly from +50 μm to −32 μm, with a change range of 82 μm and an average regulation sensitivity of 0.164 μm/KN. In contrast, the variation range of the fourth-order crown is only from +5 μm to −3 μm, with a change range of 8 μm and a regulation sensitivity of 0.016 μm/KN, which is about one-tenth of that of the secondary crown.

When WRB is −100 KN, the secondary camber is +50 μm and the fourth-order camber is +5 μm, exhibiting a clear trend of symmetrical edge wave. As WRB increases, both camber components decrease simultaneously, approaching 0 μm at WRB = 200 KN, achieving an ideal cross-sectional shape. Continuing to increase WRB to 400 KN, the secondary camber turns negative, and the fourth-order camber also turns negative, corresponding to a trend of mid-wave defects.

This difference stems from the mechanical characteristics of work roll bending deformation: the deformation of the work roll under the action of bending force mainly manifests as a simple beam bending mode, which primarily affects the secondary crown component. The fourth-order crown corresponds to a more complex roll barrel deformation mode, which requires higher-order bending moments for effective regulation, and the work roll bending force has limited regulatory capability in this regard. Under conventional rolling forces, the work roll bending force provides an effective means for achieving precise crown control. Precise control of crown can be achieved through simple linear adjustments, which is conducive to the implementation of automatic control systems.

#### 4.2.2 Influence of work roll bending force on crown under high rolling force.

**Fig 11:**
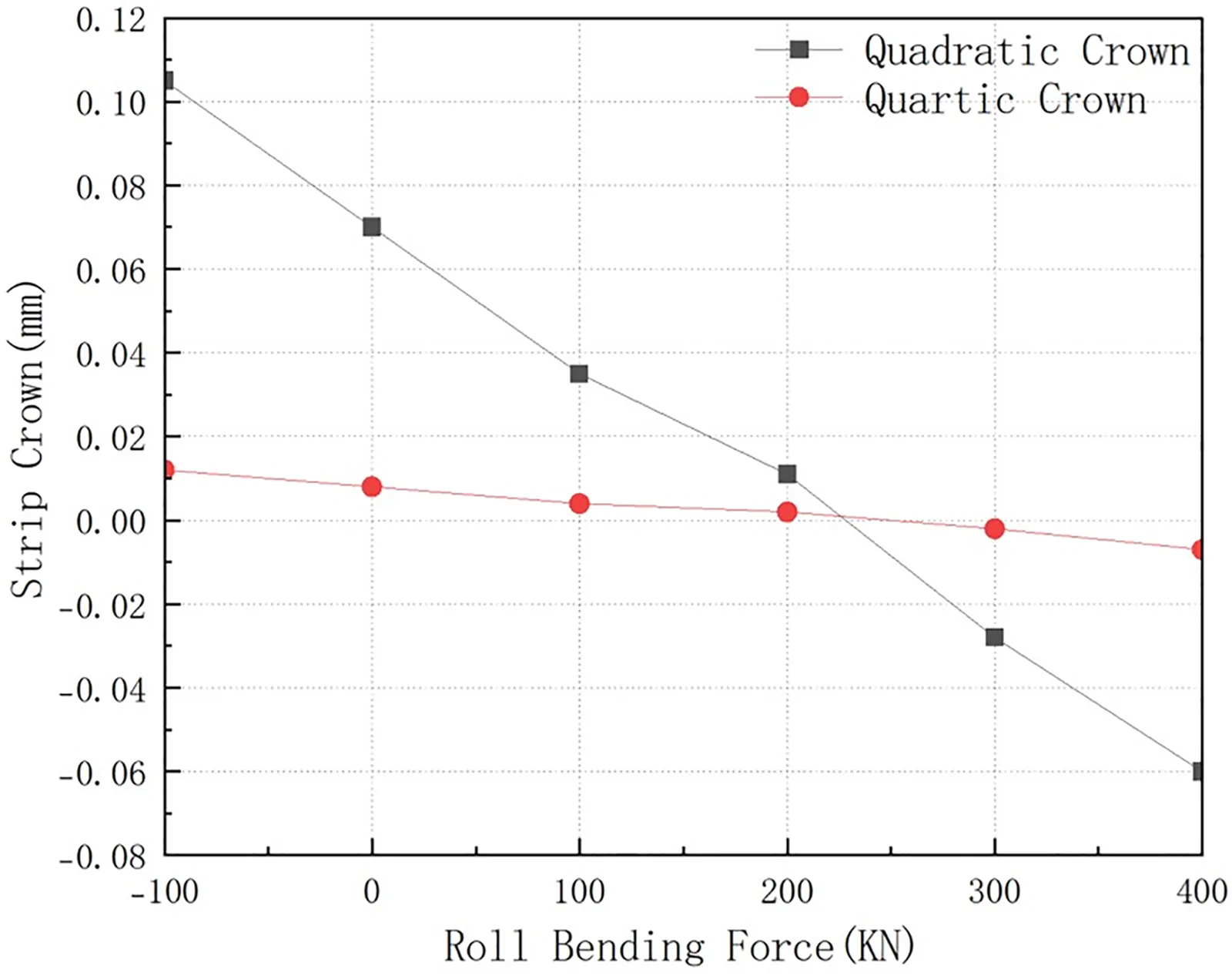
Effect of work roll bending force on strip crown under high rolling force.

Under high rolling force conditions, the control characteristics of work roll bending force on crown components change significantly, as shown in Fig 11.

Under high rolling force conditions, the work roll bending force significantly enhances the regulation capability for both types of crown components. When the WRB (Work Roll Bending force) increases from −100 KN to 400 KN, the secondary crown changes from +105 μm to −58 μm, with a total variation of 163 μm, and the regulation sensitivity increases to 0.326 μm/KN, which is twice that of conventional operating conditions. The variation range of the fourth-order crown also changes from the conventional 12 μm to −8 μm, and the regulation sensitivity increases to 0.072 μm/KN.

It is noteworthy that the WRB value corresponding to the optimal crown under high rolling force experiences a shift. Under conventional rolling force, both crown components approach 0 when WRB = 200 KN; however, under high rolling force, the secondary crown is +10 μm and the fourth-order crown is +2 μm at WRB = 200 KN, and the crown approaches 0 only when WRB ≈ 220 KN. This shift reflects the change in the equilibrium point of roll deformation caused by the increase in rolling force. Another important phenomenon is that the relative importance of the fourth-order crown increases under high rolling force. Under conventional rolling force, the maximum absolute value of the fourth-order crown is only 5 μm, which is negligible compared to the secondary crown; however, under high rolling force, the maximum value of the fourth-order crown reaches 12 μm, and its proportion in the total crown increases from 10% to 11.4%. This indicates that under high rolling force conditions, attention should be paid to the control of both crown components simultaneously. The expanded range of crown regulation under high rolling force is a favorable factor, but the increased regulation sensitivity and shift in equilibrium point add to the difficulty of control. The drastic changes in the contour map indicate the need for a more precise setting model and faster response speed.

#### 4.2.3 Influence of intermediate roll bending force on crown under conventional rolling force.

**Fig 12:**
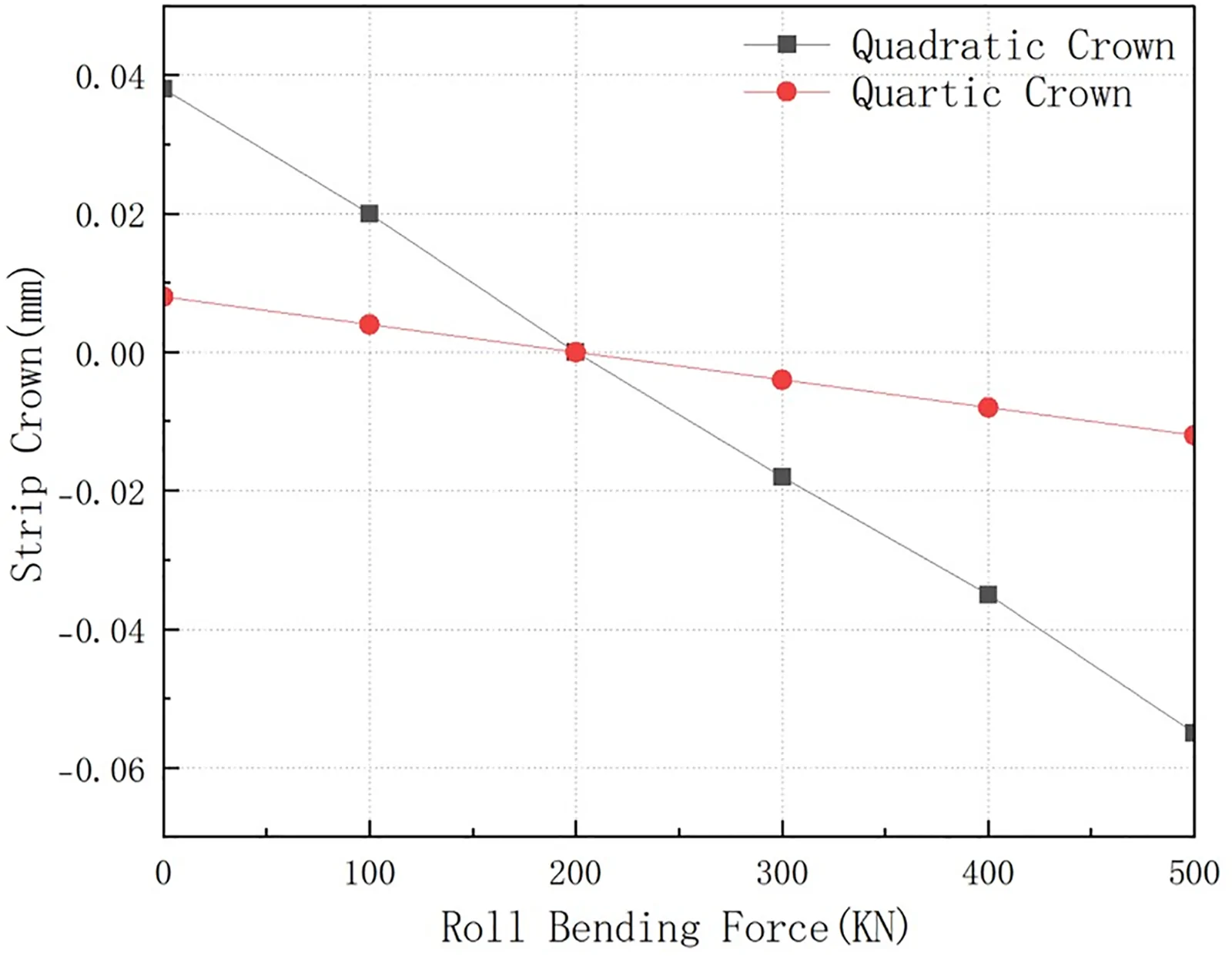
Effect of intermediate roll bending force on strip crown under conventional rolling force.

Intermediate roll bending force exhibits unique control characteristics over crown components under conventional rolling force, as shown in Fig 12.

The bending force of the intermediate roll exhibits strong regulating capabilities for both types of crown components under conventional rolling forces. When the IRB force increases from 0 KN to 500 KN, the secondary crown decreases linearly from +38 μm to −54 μm, with a change amplitude of 92 μm and a regulating sensitivity of 0.184 μm/KN. The fourth-order crown changes from +8 μm to −12 μm, with a change amplitude of 20 μm and a regulating sensitivity of 0.040 μm/KN.

Compared to the work roll bending force, the intermediate roll bending force exhibits significantly higher efficiency in regulating the fourth-order crown. The sensitivity of the work roll bending force to regulating the fourth-order crown is only 0.016 μm/KN, whereas the intermediate roll bending force achieves 0.040 μm/KN, representing a 150% improvement. This indicates that the intermediate roll bending force can effectively regulate more complex flatness defect patterns. When IRB = 0 KN, the second-order crown is +38 μm and the fourth-order crown is +8 μm, exhibiting a pronounced edge wave trend. When IRB = 200 KN, both crown components reach 0 μm, achieving ideal flatness. When IRB = 500 KN, the second-order crown decreases to −54 μm and the fourth-order crown decreases to −12 μm, exhibiting a mid-wave trend.

The efficiency of the intermediate roll bending force stems from its unique working mechanism: the bending deformation of the intermediate roll not only affects the overall bending of the work roll but also alters the distribution of contact pressure between the work roll and the intermediate roll, resulting in a more complex bending moment distribution on the surface of the work roll. This complex bending moment distribution precisely corresponds to the regulation mode required for fourth-order crown. The efficiency of the intermediate roll bending force originates from its special working mechanism. The bending deformation of the intermediate roll not only affects the overall bending of the work roll but also alters the distribution of contact pressure between the work roll and the intermediate roll, resulting in a more complex bending moment distribution on the surface of the work roll. The complex color distribution pattern in the contour plot reflects this dual regulation effect.

#### 4.2.4 Influence of intermediate roll bending force on crown under high rolling force.

**Fig 13:**
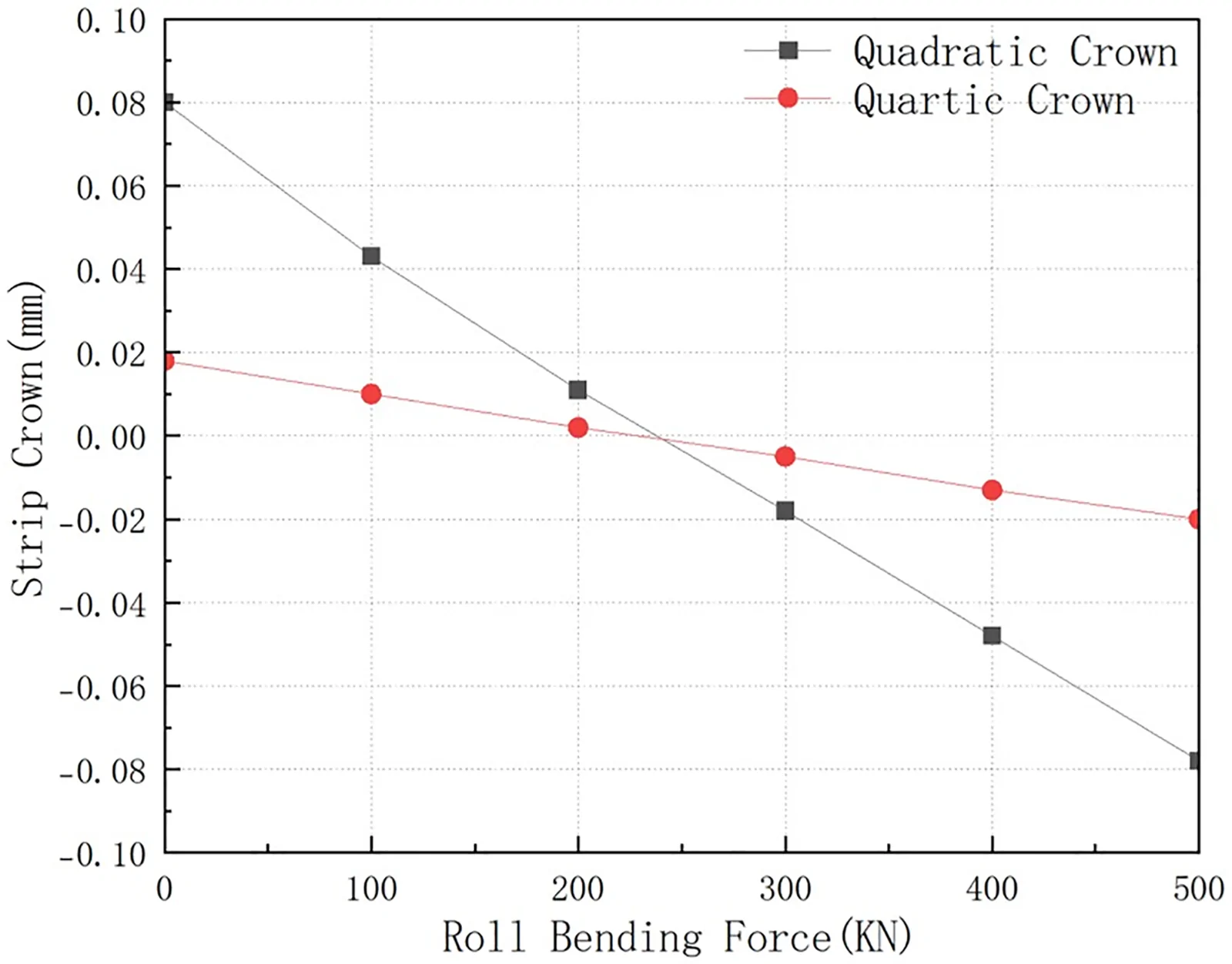
Effect of intermediate roll bending force on strip crown under high rolling force.

Under high rolling force conditions, the crown control capability of intermediate roll bending force is further enhanced, especially its control effect on quartic crown becomes more significant, as shown in Fig 13.

Under high rolling force conditions, the adjustment range of the intermediate roll bending force on the crown component is significantly expanded. When the IRB increases from 0 KN to 500 KN, the secondary crown changes from +80 μm to −78 μm, with a total variation of 158 μm and a regulation sensitivity of 0.316 μm/KN. The fourth-order crown changes from +18 μm to −20 μm, with a variation of 38 μm and a regulation sensitivity of 0.076 μm/KN.

Compared to conventional operating conditions, the sensitivity of intermediate roll bending force adjustment under high rolling force conditions has been comprehensively improved: the sensitivity of second-order camber adjustment has increased from 0.184 μm/KN to 0.316 μm/KN, and the sensitivity of fourth-order camber adjustment has increased from 0.040 μm/KN to 0.076 μm/KN. This indicates that under high rolling force conditions, intermediate roll bending force becomes a more effective means of flatness control.

An important discovery is that the IRB value corresponding to the optimal crown under high rolling force experiences a shift. Under conventional rolling force, both crown components are 0 when IRB = 200 KN; however, under high rolling force, the secondary crown is +12 μm and the fourth-order crown is +3 μm when IRB = 200 KN, and the crown approaches 0 only when IRB ≈ 150 KN. This shift is opposite to the direction of the shift in work roll bending force, reflecting the complementary characteristics of different bending forces under extreme operating conditions. Another noteworthy phenomenon is that the relative amplitude of the fourth-order crown further increases under high rolling force. Under conventional rolling force, the maximum absolute value of the fourth-order crown is 12 μm, accounting for 24% of the total crown; under high rolling force, the maximum absolute value of the fourth-order crown reaches 20 μm, accounting for 25.6% of the total crown. This indicates that as rolling force increases, the shape defect patterns tend to become more complex, and the control requirements for the fourth-order crown correspondingly increase.

### 4.3 Flatness influence analysis

Flatness is a key indicator for measuring the flatness of strip steel surface, usually expressed as relative length difference (I-unit). In order to deeply analyze the influence of bending force on flatness distribution, this section adopts the form of transverse distribution diagram to show the variation law of flatness along the width direction of strip steel under different bending force settings. This analysis method can intuitively reveal the specific patterns and locations of plate shape defects.

#### 4.3.1 Influence of work roll bending force on flatness under conventional rolling force.

Under conventional rolling force and fixed IRB, the transverse flatness distribution of the strip shows regular changes when work roll bending force varies from −100 KN to 400 KN.

**Fig 14:**
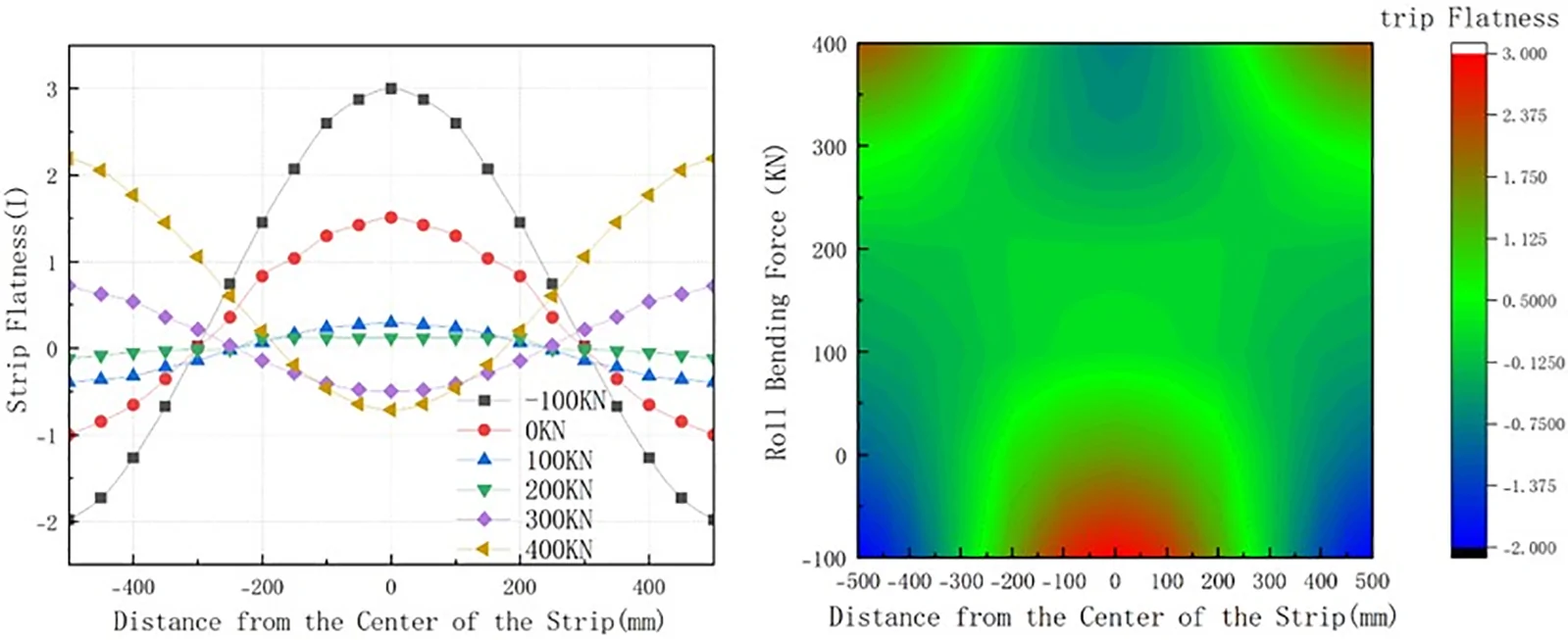
Effect of work roll bending force on strip flatness under conventional rolling force.

From Fig 14, it is clear to see the flatness distribution characteristics under different work roll bending force settings: when WRB = −100 KN, the flatness distribution exhibits a typical “edge wave” pattern, with the flatness values at the edges being higher than those in the central area, indicating that the extension of the strip steel edges is significantly greater than that of the central part. This distribution can lead to obvious waviness in the strip steel during subsequent processes.

As the WRB increases to 0 KN, the degree of side wave decreases, the flatness of the edges reduces, and the central area also decreases. When WRB = 100 KN, the flatness distribution further improves, and the overall distribution tends to be flat. At WRB = 200 KN, the flatness distribution is most ideal, with flatness values close to 0 I across the entire width, indicating uniform lateral extension and no obvious wave defects. When WRB continues to increase to 300 KN, the flatness distribution begins to exhibit a “medium wave” trend, with the flatness in the central area increasing and the edges decreasing. At WRB = 400 KN, the medium wave phenomenon becomes more pronounced.

This distribution change reflects the impact of work roll bending on the shape of the roll gap: negative bending force causes the work roll to bend towards the operating side and the driving side, resulting in a smaller roll gap at the edges than in the middle, producing edge waves; positive bending force causes the work roll to bend towards the center, resulting in a smaller roll gap in the middle than at the edges, producing center waves. The optimal flatness is achieved when WRB = 200 KN, at which point the bending moment precisely compensates for the bending deformation of the work roll caused by the rolling force. The perfect uniformity of the contour map verifies this equilibrium state.

#### 4.3.2 Influence of work roll bending force on flatness under high rolling force.

Under high rolling force conditions, the influence of work roll bending force on flatness distribution is significantly enhanced, and the amplitude of defect patterns increases noticeably.

**Fig 15:**
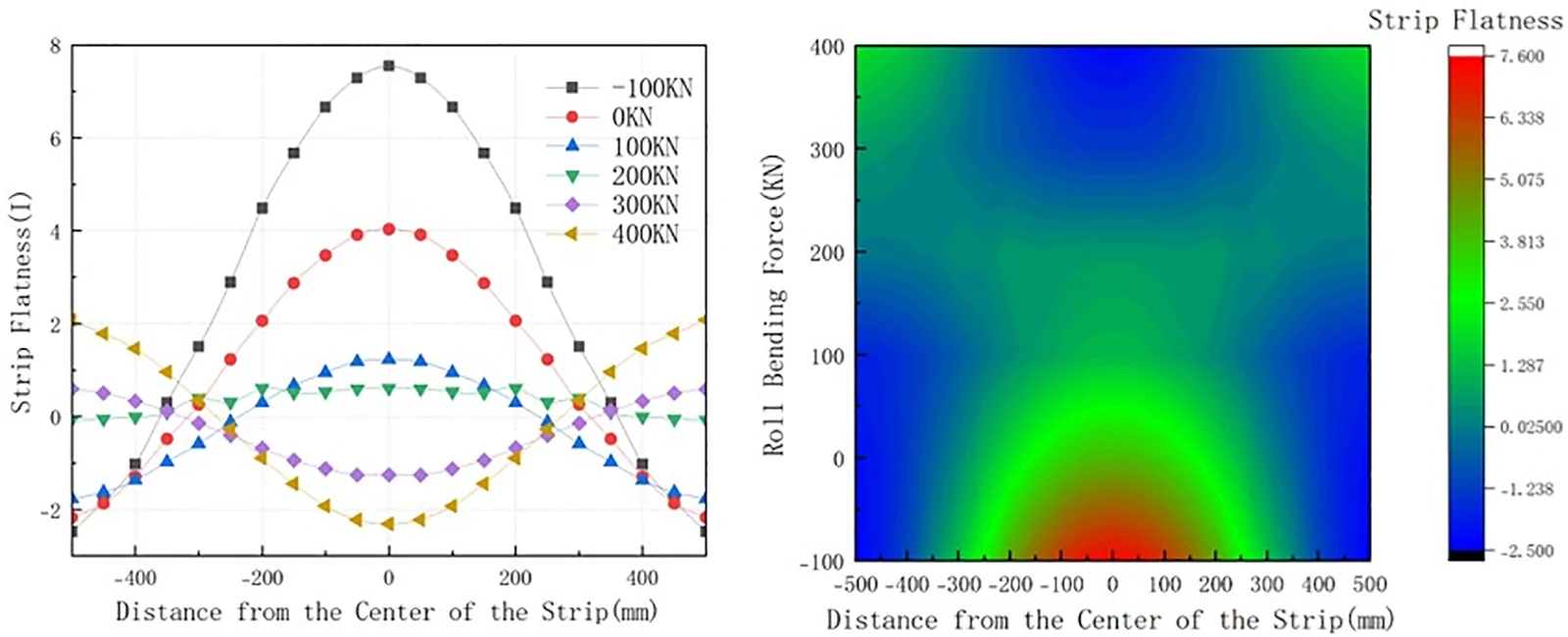
Effect of work roll bending force on strip flatness under high rolling force.

As can be seen from Fig 15, the flatness distribution under high rolling force exhibits more significant changes in characteristics: when WRB = −100 KN, the edge wave phenomenon is extremely severe, with high flatness at the edges and a higher t value in the central area, representing an increase of 130% compared to conventional conditions. Such severe edge waves can lead to production accidents such as strip steel deviation and tail swinging in actual production. When WRB = 0 KN, the degree of edge wave is alleviated, but the flatness at the edges and the central area far exceeds the acceptable range. When WRB = 100 KN, the flatness distribution is significantly improved, but it still does not reach the ideal state. It is worth noting that under high rolling force, even with the optimal setting of WRB = 200 KN, the flatness distribution is still not completely flat, with residual wave patterns at the edges. This indicates that under high rolling force conditions, it is difficult to completely eliminate shape defects solely by relying on work roll bending force. When WRB = 300 KN and 400 KN, the mid-wave phenomenon is more pronounced than under conventional conditions.

An important finding is that the “steepness” of the flatness distribution curve increases under high rolling force, indicating a more pronounced change in the defect area. This suggests that shape defects caused by high rolling force become more localized and acute, making control more challenging.

#### 4.3.3 Influence of intermediate roll bending force on flatness under conventional rolling force.

Under conventional rolling force and fixed WRB, intermediate roll bending exhibits unique control characteristics over flatness distribution.

**Fig 16:**
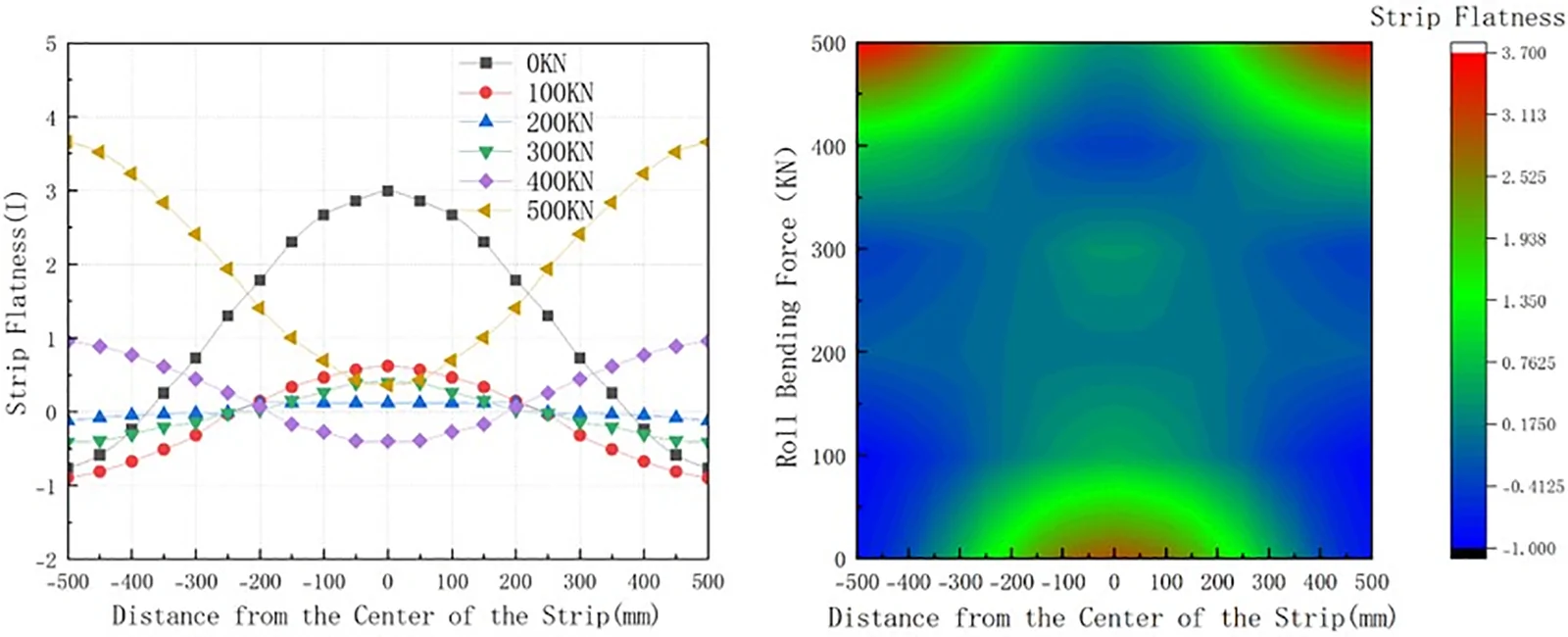
Effect of intermediate roll bending force on strip flatness under conventional rolling force.

As can be seen from Fig 16, the influence of the intermediate roll bending force on the flatness distribution under conventional rolling force exhibits the following characteristics: When IRB = 0 KN, the flatness distribution shows obvious edge wave characteristics. As IRB increases to 100 KN, the degree of edge wave significantly decreases, and the edge value drops. When IRB = 200 KN, the flatness distribution reaches its optimal state, with flatness values close to 0 I-unit across the entire width. Compared with the work roll bending force, the intermediate roll bending force provides a flatter flatness distribution near the optimal value, i.e., with less fluctuation in the central region. When IRB continues to increase to 300 KN, a mid-wave trend begins to emerge, with an increase in flatness in the central region. At IRB = 400 KN, the mid-wave phenomenon becomes more pronounced. At IRB = 500 KN, severe mid-wave occurs.

An important finding is that the bending force of the intermediate roll has a more moderate regulation effect on the flatness distribution, indicating a smoother transition from edge wave to center wave. In contrast, the bending force of the work roll causes more drastic changes in flatness. This suggests that the bending force of the intermediate roll provides a more refined flatness control capability. Another noteworthy phenomenon is that the regulation effect of the bending force of the intermediate roll is particularly significant in the quarter-section area. This is reflected in Fig 15 as smooth transitions of curves at these positions, avoiding local abrupt changes that may be caused by the bending force of the work roll.

#### 4.3.4 Influence of intermediate roll bending force on flatness under high rolling force.

Under high rolling force conditions, the control of flatness distribution by intermediate roll bending force faces new challenges but also demonstrates unique advantages.

**Fig 17:**
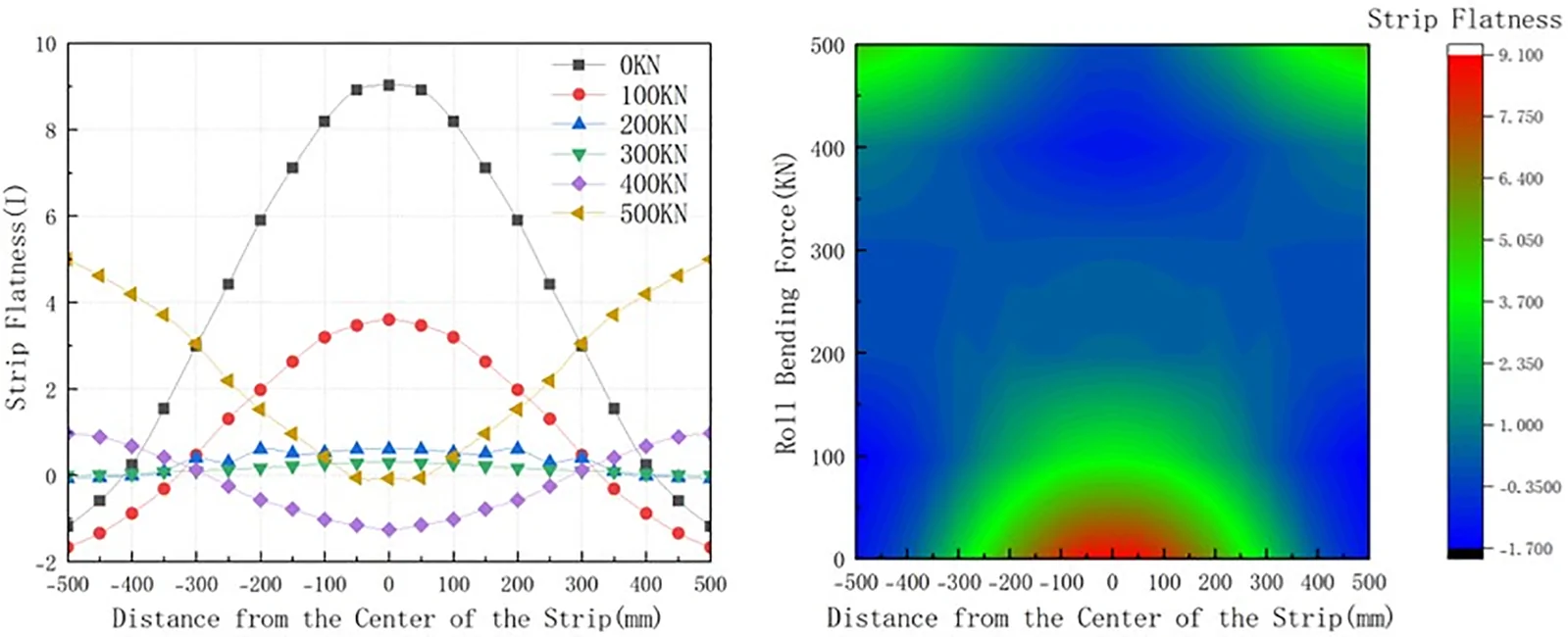
Effect of intermediate roll bending force on strip flatness under high rolling force.

As can be seen from Fig 17, the flatness control characteristics of the intermediate roll bending force under high rolling force exhibit the following important features: When IRB = 0 KN, the edge wave phenomenon is extremely severe. Compared with the work roll bending force, the edge wave amplitude of the intermediate roll bending force under extreme negative values is slightly lower, indicating that its control characteristics are more moderate. As IRB increases to 100 KN, the flatness distribution is significantly improved, and the edge values decrease, but they are still far beyond the acceptable range. When IRB = 200 KN, a relatively optimal state is achieved, but residual waves still exist at the edges. It is worth noting that under high rolling force, the intermediate roll bending force does not achieve a completely flat distribution at IRB = 200 KN, which contrasts with the perfect control under conventional operating conditions. This indicates that the roll system deformation caused by high rolling force has exceeded the independent compensation capability of the intermediate roll bending force. When IRB increases to 300 KN and 400 KN, the mid-wave phenomenon gradually emerges. When IRB = 500 KN, severe mid-waves occur.

An important finding is that the control effect of the intermediate roll bending force on the quarter-section is more significant under high rolling force. In Fig 16, all curves exhibit good smoothing characteristics in the quarter-section, avoiding the local stress concentration that may be caused by the work roll bending force. Another noteworthy phenomenon is that the optimal value region of the intermediate roll bending force widens under high rolling force. Although IRB = 200 KN is the theoretical optimal point, IRB within the range of 150–250 KN can provide a relatively acceptable flatness distribution. This provides greater flexibility for parameter adjustment in actual production.

### 4.4 Rolling force distribution influence analysis

The uniformity of the lateral distribution of rolling force directly affects the strip shape quality, work roll wear, and rolling stability. To deeply analyze the mechanism of the influence of bending force on the distribution of rolling force, this section adopts the form of lateral distribution diagrams to show the characteristics of rolling force distribution along the strip steel width under different bending force settings. This analysis method can visually reveal the unevenness of load distribution and the compensating effect of bending force.

#### 4.4.1 Influence of work roll bending force on rolling force distribution under conventional rolling force.

Under conventional rolling force and fixed IRB, work roll bending force has a significant control effect on rolling force distribution.

**Fig 18:**
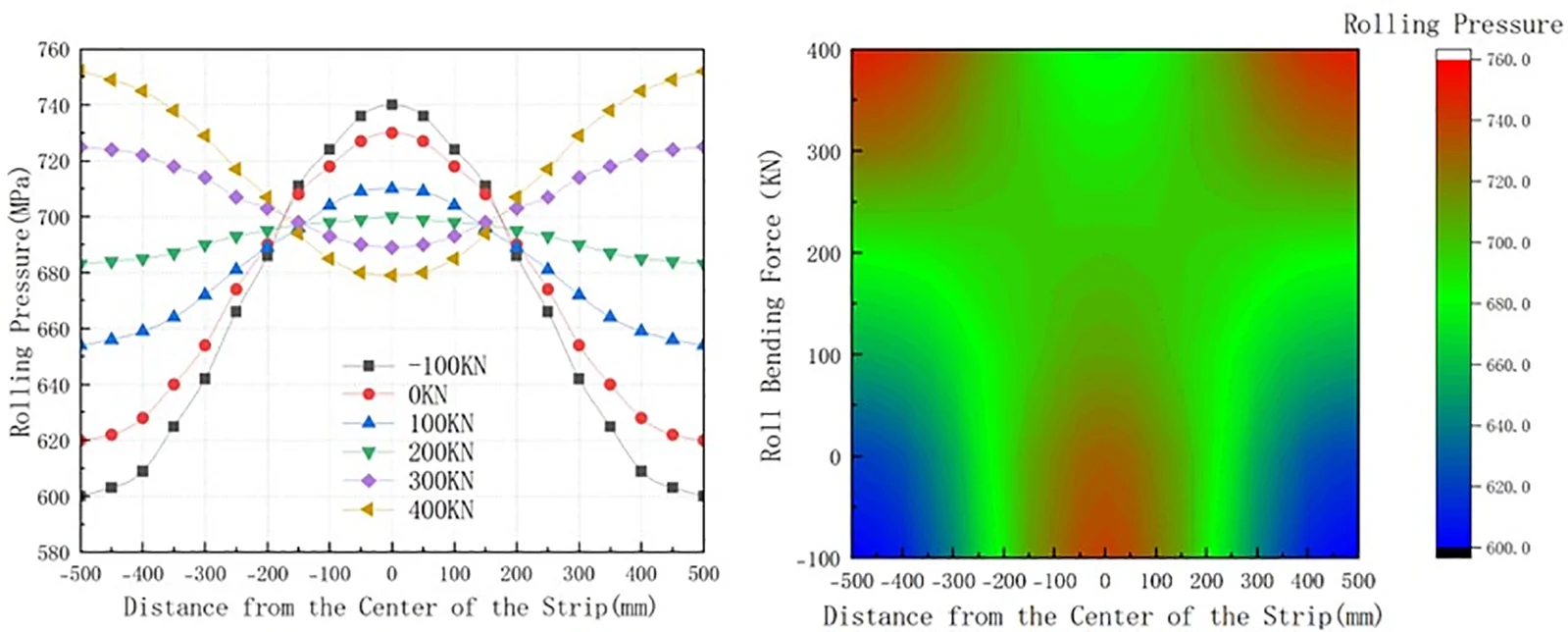
Effect of work roll bending force on rolling force distribution under conventional rolling force.

From Fig 18, it is clear to see the distribution characteristics of rolling force under different work roll bending force settings: when WRB = −100 KN, the rolling force distribution exhibits a severe edge concentration phenomenon, with rolling forces of 600KN at both ends and 740KN in the central area, resulting in an unevenness of 81%. This distribution can lead to accelerated uneven wear of the work roll, seriously affecting the quality of the plate shape.

As the WRB (Work Roll Bending) increases to 0 KN, the edge concentration phenomenon eases, with the edge rolling force rising to 620 KN and the center area to 730 KN, and the unevenness decreases to 70%. When WRB = 100 KN, the distribution further improves, with the edge value reaching 650 KN and the unevenness decreasing to 33.1%. At WRB = 200 KN, the rolling force distribution is the most uniform, with the rolling force across the entire width remaining at around 690 KN, and the unevenness approaching 0. This indicates that under this setting, the bending deformation of the work roll compensates for the roll system deformation caused by the rolling force. When WRB continues to increase to 300 KN, a central concentration trend begins to emerge, with the center area rolling force reaching 690 KN and the edge to 720 KN. At WRB = 400 KN, the central concentration phenomenon becomes more pronounced, with the center area rolling force reaching 680 KN and the edge to 750 KN, and the unevenness is 30.8%.

This distribution change reflects the impact of work roll bending on the roll gap stiffness distribution: negative bending force causes the work roll to bend towards both sides, leading to an increase in actual stiffness at the edges and a concentration of rolling force; positive bending force causes the work roll to bend towards the center, leading to an increase in actual stiffness in the middle and a concentration of rolling force. A uniform distribution of rolling force not only improves the quality of plate shape but also reduces uneven wear of the work roll, extending equipment life. The uniform distribution of the contour map provides intuitive guidance for equipment protection.

#### 4.4.2 Influence of work roll bending force on rolling force distribution under high rolling force.

Under high rolling force conditions, the control of rolling force distribution by work roll bending force faces greater challenges.

**Fig 19:**
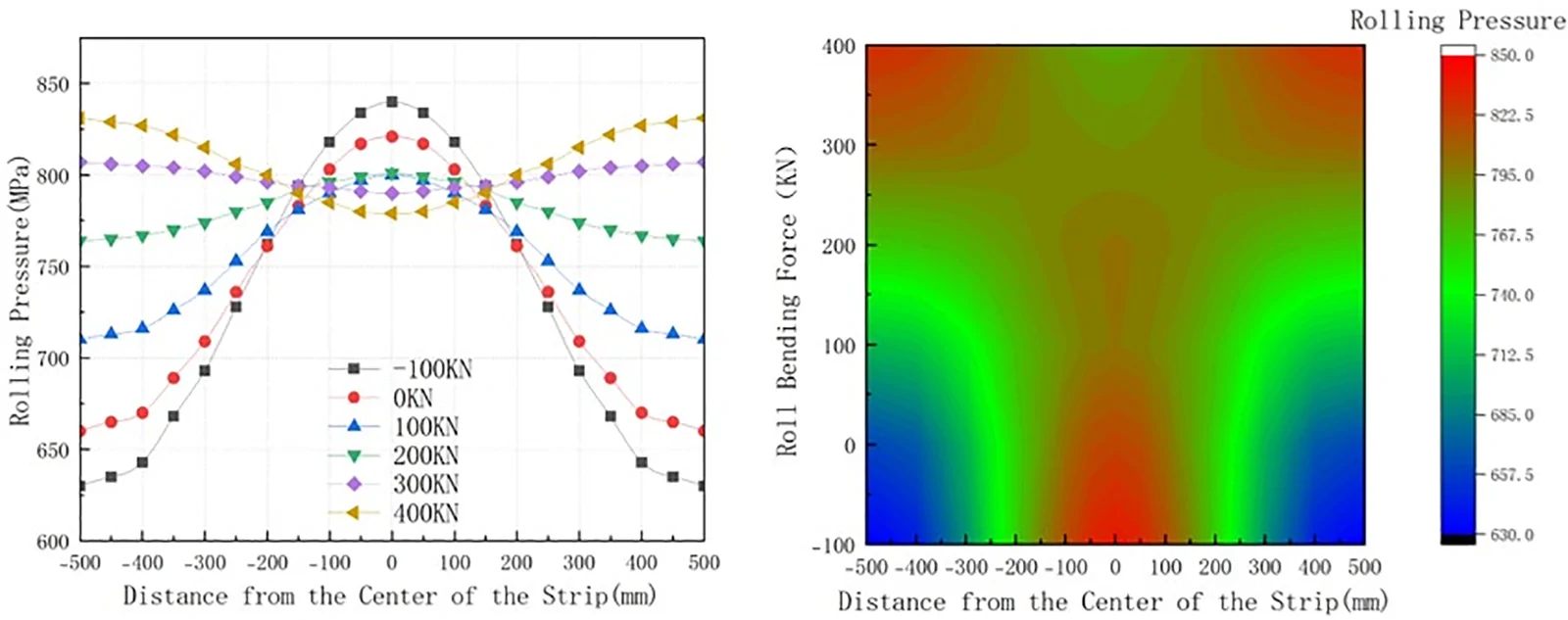
Effect of work roll bending force on rolling force distribution under high rolling force.

As can be clearly seen from Fig 19, the distribution of rolling force under high rolling force exhibits more significant changes in characteristics: when WRB = −100 KN, the edge concentration phenomenon is extremely severe, with the edge rolling force reaching 640 KN, an increase of 68.4% compared to conventional conditions, while the center region has a rolling force of 845 KN, with a non-uniformity of 88.2%. This extreme load distribution poses a serious threat to equipment safety. When WRB = 0 KN, the degree of edge concentration is alleviated, but the edge rolling force is still 655 KN, the center region has a rolling force of 830 KN, and the non-uniformity is 61.1%. When WRB = 100 KN, the distribution is significantly improved, with the edge value being 715 KN and the non-uniformity reduced to 25%. It is worth noting that under high rolling force conditions, even with the optimal setting of WRB = 200 KN, the rolling force distribution is still not completely uniform, with a residual concentration of about 760 KN at the edge and 800 KN in the center region, with a non-uniformity of 8.7%. This indicates that under high rolling force conditions, it is difficult to completely eliminate uneven load distribution solely by relying on work roll bending force.

An important discovery is that the “steepness” of the rolling force distribution curve increases under high rolling force, indicating a more pronounced change in the load-concentrated area. This suggests that the deformation of the roll system caused by high rolling force becomes more localized, making control more challenging. Another noteworthy phenomenon is the shift in the WRB value corresponding to the optimal rolling force distribution under high rolling force. Under conventional rolling force, the distribution is most uniform at WRB = 200 KN; however, under high rolling force, significant unevenness remains at WRB = 200 KN, and it is only when WRB ≈ 250 KN that the distribution reaches a relatively optimal state.

#### 4.4.3 Influence of intermediate roll bending force on rolling force distribution under conventional rolling force.

Under conventional rolling force and fixed WRB, intermediate roll bending exhibits unique control characteristics over rolling force distribution.

**Fig 20:**
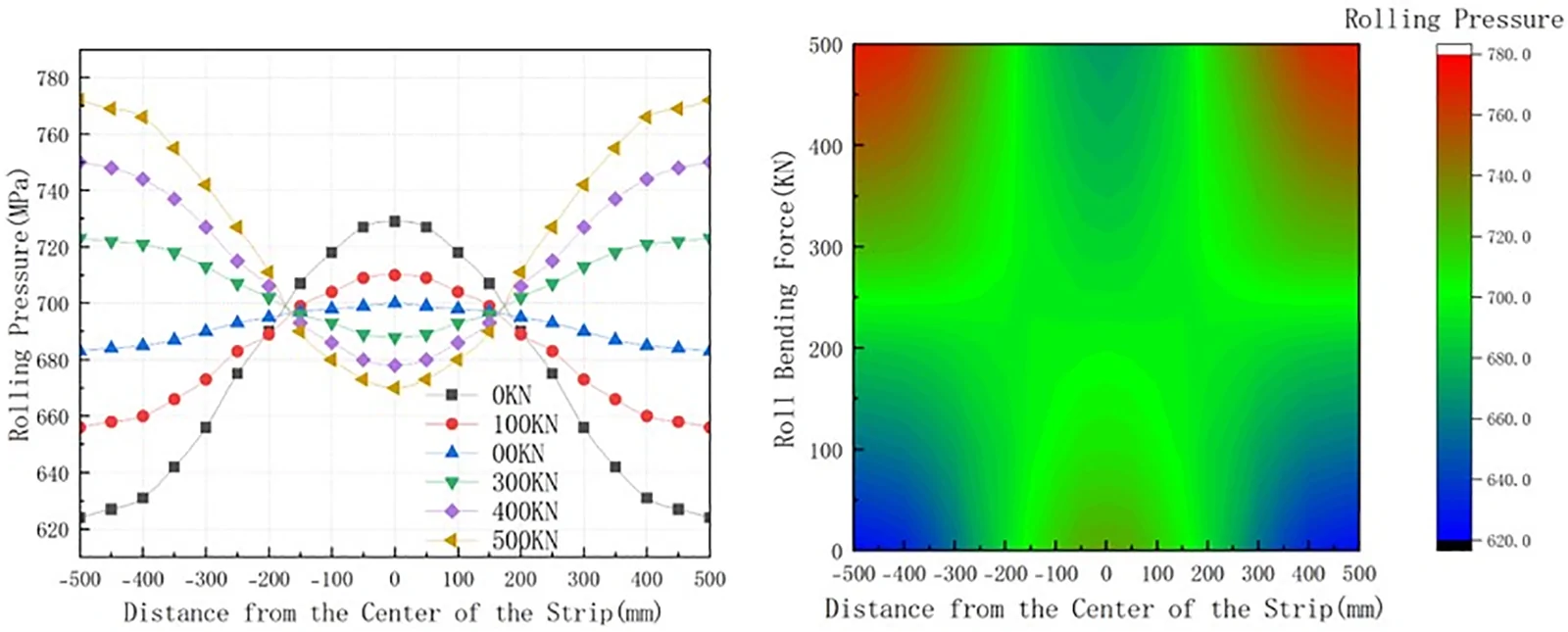
Effect of intermediate roll bending force on rolling force distribution under conventional rolling force.

As can be clearly seen from Fig 20, the influence of the intermediate roll bending force on the distribution of rolling force under conventional rolling force exhibits the following characteristics: When IRB = 0 KN, the distribution of rolling force shows a significant edge concentration feature, with the edge rolling force being 625 KN and the central area rolling force being 730 KN, resulting in an unevenness of 58.3%. As IRB increases to 100 KN, the degree of edge concentration significantly decreases, with the edge value being 675 KN and the unevenness dropping to 30.8%. When IRB = 200 KN, the distribution of rolling force reaches its optimal state, with the rolling force across the entire width remaining approximately at 690 KN and the unevenness nearing 0. Compared to the work roll bending force, the intermediate roll bending force provides a “smoother” distribution of rolling force near the optimal value, meaning there is less fluctuation in the transition zone. When IRB continues to increase to 300 KN, a central concentration trend begins to emerge, with the central area rolling force being 690 KN and the edge rolling force being 720 KN. When IRB = 400 KN, the central concentration phenomenon becomes more pronounced, with the central area rolling force being 680 KN and the edge rolling force being 750 KN. When IRB = 500 KN, severe central concentration occurs, with the central area rolling force being 670 KN and the edge rolling force being 770 KN, resulting in an unevenness of 50%.

An important discovery is that the regulation of rolling force distribution by the intermediate roll bending force is more gradual, indicating a smoother transition from edge-concentrated to mid-concentrated distribution. In contrast, the change in rolling force distribution caused by the work roll bending force is more “steep.” This suggests that the intermediate roll bending force provides a more stable load distribution control capability. Another noteworthy phenomenon is that the regulation effect of the intermediate roll bending force is particularly significant in the quarter-section area. This is reflected in Fig 19 as smooth transitions of the curves at these positions, avoiding local load mutations that may be caused by the work roll bending force.

#### 4.4.4 Influence of Intermediate Roll Bending Force On Rolling Force Distribution Under High Rolling Force.

Under high rolling force conditions, the control of rolling force distribution by intermediate roll bending force faces new challenges but also demonstrates unique advantages.

**Fig 21:**
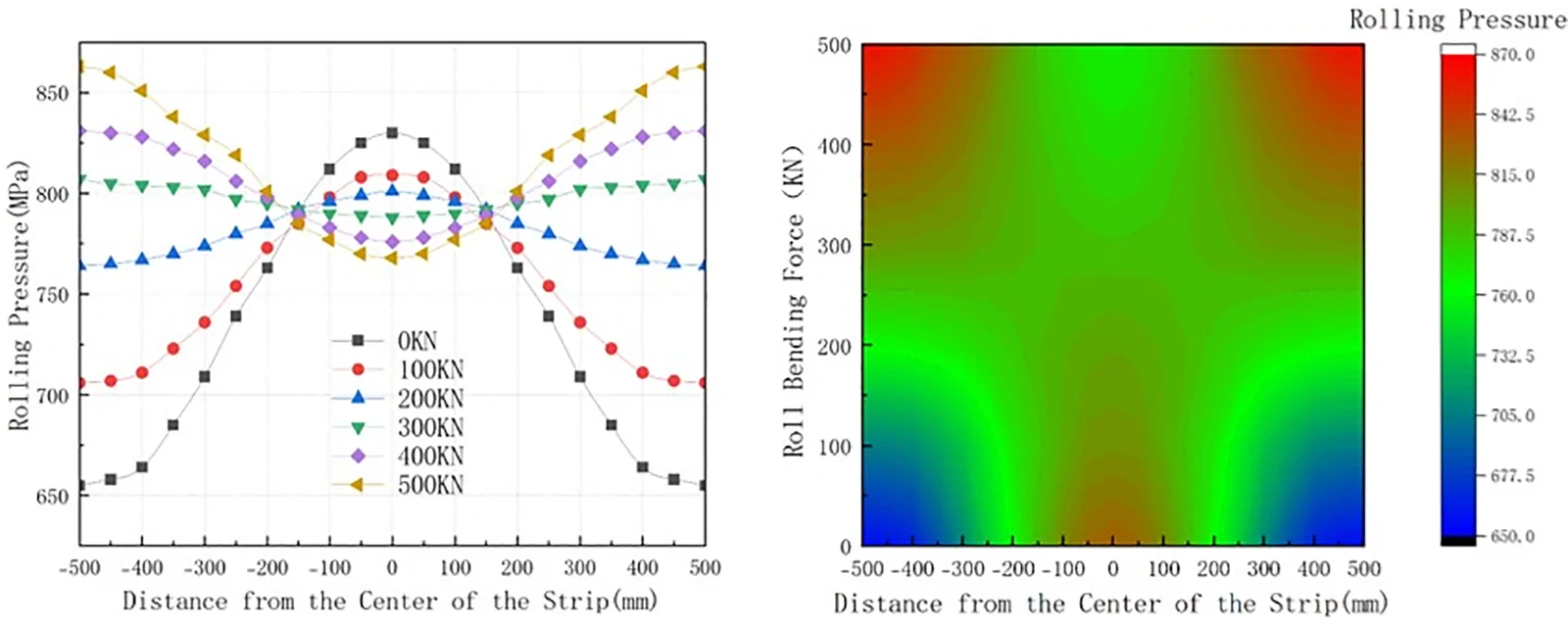
Effect of intermediate roll bending force on rolling force distribution under high rolling force.

As can be clearly seen from Fig 21, the regulation characteristics of rolling force distribution under high rolling force with intermediate roll bending force exhibit the following important features: When IRB = 0 KN, the edge concentration phenomenon is severe, with edge rolling force of 650 KN and central region rolling force of 835 KN, resulting in a non-uniformity of 66.7%. Compared with the work roll bending force, the edge concentration amplitude of the intermediate roll bending force under extreme negative values is slightly lower, indicating that its regulation characteristics are more moderate. As IRB increases to 100 KN, the rolling force distribution is significantly improved, with edge values of 705 KN and non-uniformity reduced to 30%. When IRB = 200 KN, a relatively optimal state is achieved, but there is still a residual concentration of about 760 KN at the edges, with central region rolling force of 800 KN and non-uniformity of 8.7%. It is worth noting that under high rolling force, the intermediate roll bending force does not achieve completely uniform distribution at IRB = 200 KN, which contrasts with the perfect control under conventional operating conditions. This indicates that the roll system deformation caused by high rolling force has exceeded the independent compensation capability of the intermediate roll bending force.

An important finding is that the control effect of the intermediate roll bending force on the quarter-section is more significant under high rolling force. In Fig 21, all curves exhibit good smoothing characteristics in the quarter-section, avoiding the sudden local load changes that may be caused by the work roll bending force. Another noteworthy phenomenon is that the optimal value region of the intermediate roll bending force widens under high rolling force. Although IRB = 200 KN is the theoretical optimal point, IRB within the range of 150–250 KN can provide a relatively acceptable rolling force distribution. This provides greater flexibility for parameter adjustment in actual production.

## 5. Bending force optimization under high rolling force conditions

### 5.1 Bending force optimization strategy design

As mentioned earlier, extreme rolling force can lead to a significant increase in roll deformation, rendering the effectiveness of flatness control strategies designed for conventional operating conditions sharply reduced or even ineffective. The purpose of this section is to propose a set of bending force optimization methods tailored for high rolling force conditions, in order to effectively compensate for harmful deformation, stabilize, and enhance shape quality.

Optimizing the bending force is not simply about increasing the set value of the bending force, but rather a systematic engineering project with multiple objectives and strong constraints, based on a profound understanding of the process mechanism. Its core idea is to generate an accurate compensating roll profile that is opposite to the harmful roll gap shape distortion caused by the rolling force, by optimizing the combination of the intermediate roll bending force and its setting, so as to restore the actual roll gap to be straight.

Strip shape optimization is a typical multi-objective optimization problem, which requires simultaneous consideration of the following two core objectives: Optimal flatness: This is the primary objective. It aims to minimize the relative length difference of the strip steel outlet cross-section. The objective function can be defined as:


$$\begin{array}{c} min\left(\sum_{i=1}^{n}{\left|\Delta L_{i}\right|}^{\text{2}}\right) \end{array}$$

Where, $\Delta L_{i}$ is the relative length difference of the i-th fiber in the cross-section, n is the number of divided strips. In practice, it is often simplified to minimizing the maximum wave height of the strip or the variance of residual stress.

Minimum Transverse Thickness Deviation: While ensuring flatness, make the exit thickness distribution as close as possible to the target crown (usually zero crown or slight positive crown). The objective function can be defined as:


$$\begin{array}{c} min\left(\sqrt{\frac{\text{1}}{m}\sum_{j=1}^{m}{\left(h_{j}-h_{target}\right)}^{\text{2}}}\right) \end{array}$$

Where, $h_{j}$ is the measured thickness at point j in the cross-section, $h_{target}$ is the target thickness at that point, m is the number of measurement points.

In practical optimization, two objectives are often combined into a single comprehensive objective function, with priorities balanced by assigning weights. Under extreme rolling conditions, the weight of the flatness objective is usually higher, as severe waviness is a sign of direct scrap.

Optimization method: Directly invoking the high-precision ABAQUS finite element model for optimization search incurs extremely high computational costs, making it impractical for engineering applications. Therefore, adopting a surrogate model optimization strategy is an efficient and feasible solution.

Within the feasible region of bending force, Latin hypercube sampling is employed to obtain samples. Gaussian process regression, radial basis function neural network, and other methods are utilized to construct surrogate models. NSGA-II or particle swarm optimization algorithms are applied for global optimization. Finally, the accuracy and reliability of the optimization results are ensured through finite element verification and adaptive update mechanisms. This ensures uniform distribution of samples in space, reflecting the characteristics of the system to the greatest extent with fewer sampling points. For each sample point, the ABAQUS finite element model is run to obtain the corresponding output responses, namely the flatness index and thickness deviation index. This will constitute the dataset for training the surrogate model. Using the dataset obtained from sampling, a mathematical approximation model connecting input and output is constructed. Optimization algorithm search: Based on the accurate surrogate model, an efficient optimization algorithm is employed to search within the feasible region of bending force, seeking the optimal solution that satisfies the constraints and minimizes the comprehensive objective function. The optimal bending force combination predicted by the surrogate model is substituted into the complete ABAQUS finite element model for verification, confirming the improvement effect on plate shape. If the accuracy is insufficient, sampling points can be added near this optimal solution, the surrogate model is updated, and iterative optimization is performed.

Let the bending force vector to be optimized be denoted as $x={\left[x_{\text{1}},x_{\text{2}},...,x_{d}\right]}^{T}\in {\mathbb{R}}^{d}$, where d represents the number of independent bending force segments. Its feasible region is defined as follows:


$$\begin{array}{c} x=\left\{x\right|x_{i}^{min}\leq x_{i}\leq x_{i}^{max},i=1,...,d\} \end{array}$$

To maximize the reflection of system characteristics with limited samples, N sample points $\{X^{\left(j\right)}{\}}_{j=1}^{N}$ are generated using Latin Hypercube Sampling (LHS). LHS divides each dimension into N non-overlapping intervals with equal probability, and randomly combines the intervals of each dimension to ensure that the samples are uniformly distributed within the feasible region. Its mathematical expression is: for the i dimension, randomly permute {1,...,N} to obtain $\pi_{i}$, and let the i dimension of the sample j take the value:


$$\begin{array}{c} x_{i}^{\left(j\right)}=x_{i}^{min}+\frac{\pi_{i}\left(j\right)-0.5}{N}(x_{i}^{max}-x_{i}^{min}) \end{array}$$

This strategy ensures that the projections of sample points in each dimension are uniformly covered, avoiding undersampling or clustering.

For each sampling point $x^{\left(j\right)}$, run the ABAQUS finite element model and extract two key output responses:

Flatness index $f_{\text{1}}\left(x\right)$: Typically, it represents the root mean square of residual stress or flatness error (I-unit) along the length direction of the strip steel.

Thickness deviation index $f_{\text{2}}\left(x\right)$: The integral or maximum value of the absolute value of the difference between the actual thickness and the target thickness along the width direction.

Define the output response vector $y(x)=[f_{\text{1}}\left(x\right),f_{\text{2}}(x){]}^{T}$. The dataset is denoted as:


$$\begin{array}{c} D=\{(x^{\left(j\right)},y^{\left(j\right)})j=1,...,N\} \end{array}$$

Two complementary surrogate models are employed to approximate the input-output mapping, and the superior one is selected through cross-validation.

Gaussian Process assumes that the output function y(x) follows a Gaussian Process (GP):


$$\begin{array}{c} y\left(x\right)\varsigma p\left(m\left(x\right),k\left(x,x^{\prime }\right)\right) \end{array}$$

The mean function m(x) often takes a constant or a linear function, and the covariance function (kernel function) adopts a squared exponential kernel:


$$\begin{array}{c} k(x,x^{\prime })=\sigma_{f}^{\text{2}}exp(-\frac{\text{1}}{\text{2}}\sum_{i=1}^{d}\frac{(x_{i}-x_{i}^{\prime }{)}^{\text{2}}}{l_{i}^{\text{2}}})+\sigma_{n}^{\text{2}}\delta_{x,x^{\prime }} \end{array}$$

The hyperparameters $\sigma_{f}^{\text{2}}$ (signal variance), $l_{i}^{\text{2}}$ (length scale), and $\sigma_{n}^{\text{2}}$ (noise variance) are estimated by maximizing the log-marginal likelihood. Given a new input $x^{*}$, the predicted mean and variance are:


$$\begin{array}{c} \mu \left(x^{*}\right)=k_{*}^{T}K^{-1}y,\sigma^{\text{2}}\left(x^{*}\right)=k\left(x^{*},x^{*}\right)-k_{*}^{T}K^{-1}k_{*} \end{array}$$

Where K is the Gram matrix of training points, $k_{*}=[k(x^{*},x^{\left(1\right)}),...,k(x^{*},x^{\left(N\right)}){]}^{T}$

The output of RBFNN is a linear combination of radial basis functions:


$$\begin{array}{c} {\hat{y}}_{k}\left(x\right)=\sum_{m=1}^{M}w_{km}\emptyset (\|x-c_{m}\|),k=1,2 \end{array}$$

Where $\emptyset \left(r\right)=exp(-\beta r^{\text{2}})$ represents the Gaussian radial basis function, $c_{m}$ denotes the m-th center (which can be obtained through clustering from the samples), and M stands for the number of hidden layer nodes. The weight $w_{km}$ is solved through least squares or regularization:


$$\begin{array}{c} \underset{W}{min}{\sum_{j=1}^{N}\|y^{\left(j\right)}-\hat{y}\left(x^{\left(j\right)}\right)\|}^{\text{2}}+\lambda {\|W\|}^{\text{2}} \end{array}$$

Here, W represents the weight parameter matrix, $x^{\left(j\right)}$ represents the feature vector of the j-th sample, and $y^{\left(j\right)}$ is the true label of the j-th sample.

Within the region where the accuracy of the surrogate model is credible, seek the bending force combination that minimizes the comprehensive objective function. Define the comprehensive objective function as follows:


$$\begin{array}{c} J\left(x\right)=\alpha \cdot \frac{f_{\text{1}}\left(x\right)}{f_{\text{1}}^{nom}}+\left(1-\alpha \right)\cdot \frac{f_{\text{2}}\left(x\right)}{f_{\text{2}}^{nom}},\alpha \in \left[1,2\right] \end{array}$$

Where $f_{\text{1}}^{nom},f_{\text{2}}^{nom}$ is the reference value under nominal operating conditions, used for dimensionless normalization. The constraints include the feasible region x of the bending force and possible process constraints that may be introduced:


$$\begin{array}{c} g\left(x\right)\leq 0 \end{array}$$

Optimization problem:


$$\begin{array}{c} x^{*}=argminJ\left(x\right)s.t.g\left(x\right)\leq 0 \end{array}$$

The following two multi-objective/single-objective optimization algorithms are employed for solving:

NSGA II: Applicable to multi-objective scenarios (simultaneously optimizing $f_{\text{1}}$ and $f_{\text{2}}$). Based on rapid non-dominated sorting and crowding distance, it generates the Pareto front. Its iterative formula includes: crossover (simulated binary crossover SBX) and polynomial mutation.

Particle Swarm Optimization (PSO): used for the single-objective comprehensive function J(x). The velocity and position of particle i are updated as follows:


$$\begin{array}{c} \begin{array}{c} v_{i}\left(t+1\right)=wv_{i}\left(t\right)+c_{\text{1}}r_{\text{1}}\left(P_{i}^{best}-x_{i}\left(t\right)\right)+c_{\text{2}}r_{\text{2}}\left(g^{best}-x_{i}\left(t\right)\right)\\ x_{i}\left(t+1\right)=x_{i}\left(t\right)+v_{i}\left(t+1\right) \end{array} \end{array}$$

Where w represents the inertia weight, and $c_{\text{1}},c_{\text{2}}$ denotes the learning factor, $r_{\text{1}},r_{\text{2}}U\left(0,1\right)$

Substitute the optimal solution $x^{*}$ predicted by the surrogate model into the ABAQUS full-order finite element model to calculate the true response $y_{FEM}\left(x^{*}\right)$. Define the relative prediction error:


$$\begin{array}{c} e_{k}=\frac{\left|{\hat{y}}_{k}\left(x^{*}\right)-y_{FEM,k}\left(x^{*}\right)\right|}{y_{FEM,k}\left(x^{*}\right)},k=1,2 \end{array}$$

If $max\left(e_{\text{1}},e_{\text{2}}\right)>\varepsilon$ (ε is a preset threshold, such as 5%), initiate adaptive updating:

Add $n_{new}$ new sample points within the $x^{*}$ neighborhood (such as LHS local densification or based on the maximum prediction variance criterion); incorporate these new points into the training set D, retrain the surrogate model; and perform optimization → verification again until the accuracy requirements are met.

The iterative process can be formalized as follows:


$$\begin{array}{c} D_{t+1}=D\cup \left\{x_{new}^{\left(1\right)},...,x_{new}^{\left(new\right)}\right\},x_{new}^{\left(i\right)}\in N\left(x_{t}^{*},\delta \right) \end{array}$$

$N\left(x_{t}^{*},\delta \right)$ represents the hypercube neighborhood centered at the current optimal solution with a radius of δ.

**Fig 22:**
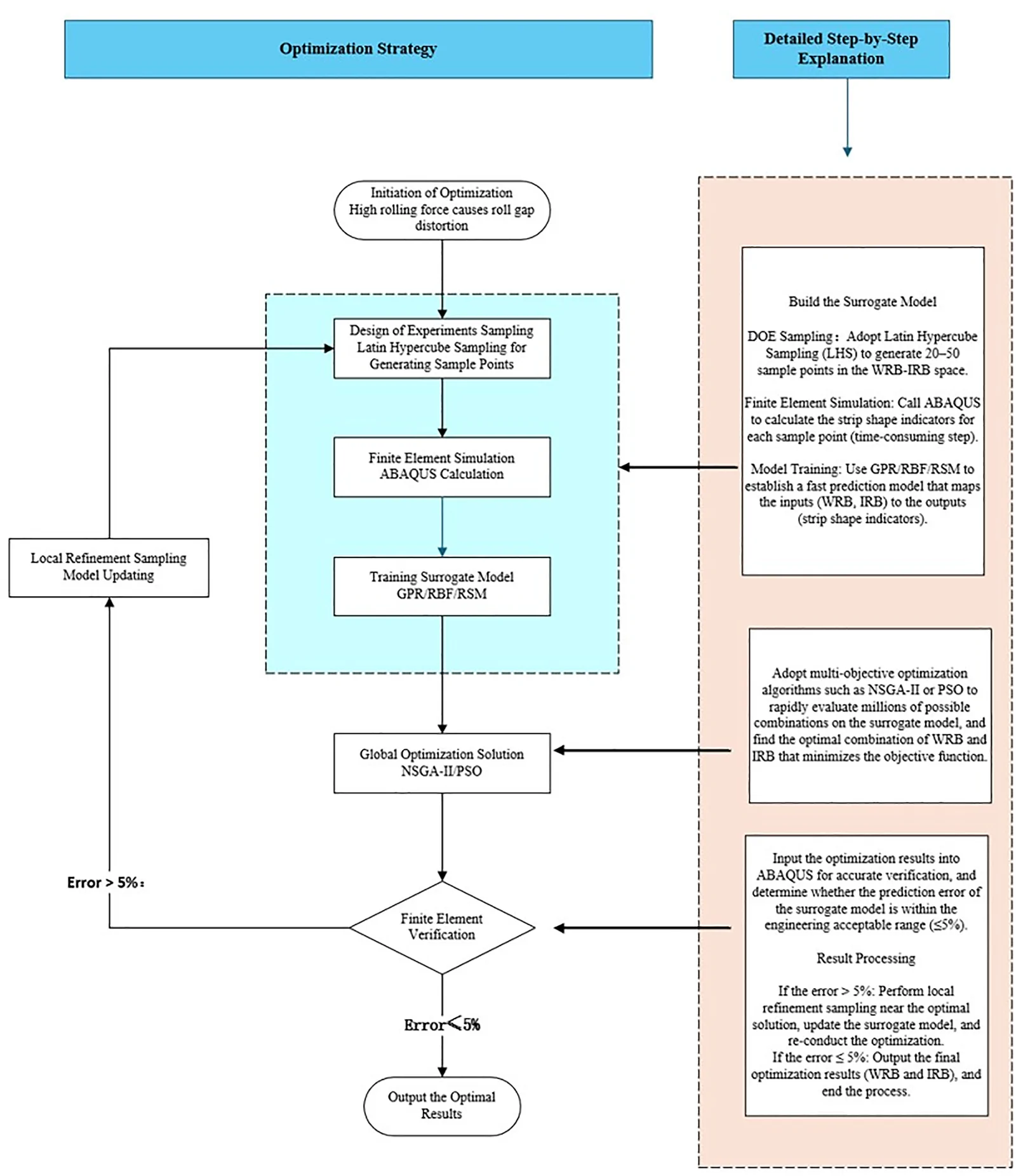
Flow chart of roll bending force optimization strategy.

The bending roller force optimization strategy flowchart is shown in Fig 22.

### 5.2 Optimization effect comparison

#### 5.2.1 Thickness optimization effect.

Thickness uniformity is a fundamental indicator for measuring rolling process stability and product quality. Under high rolling force conditions, through the implementation of the bending force optimization strategy, thickness control effectiveness has achieved significant improvement.

**Fig 23:**
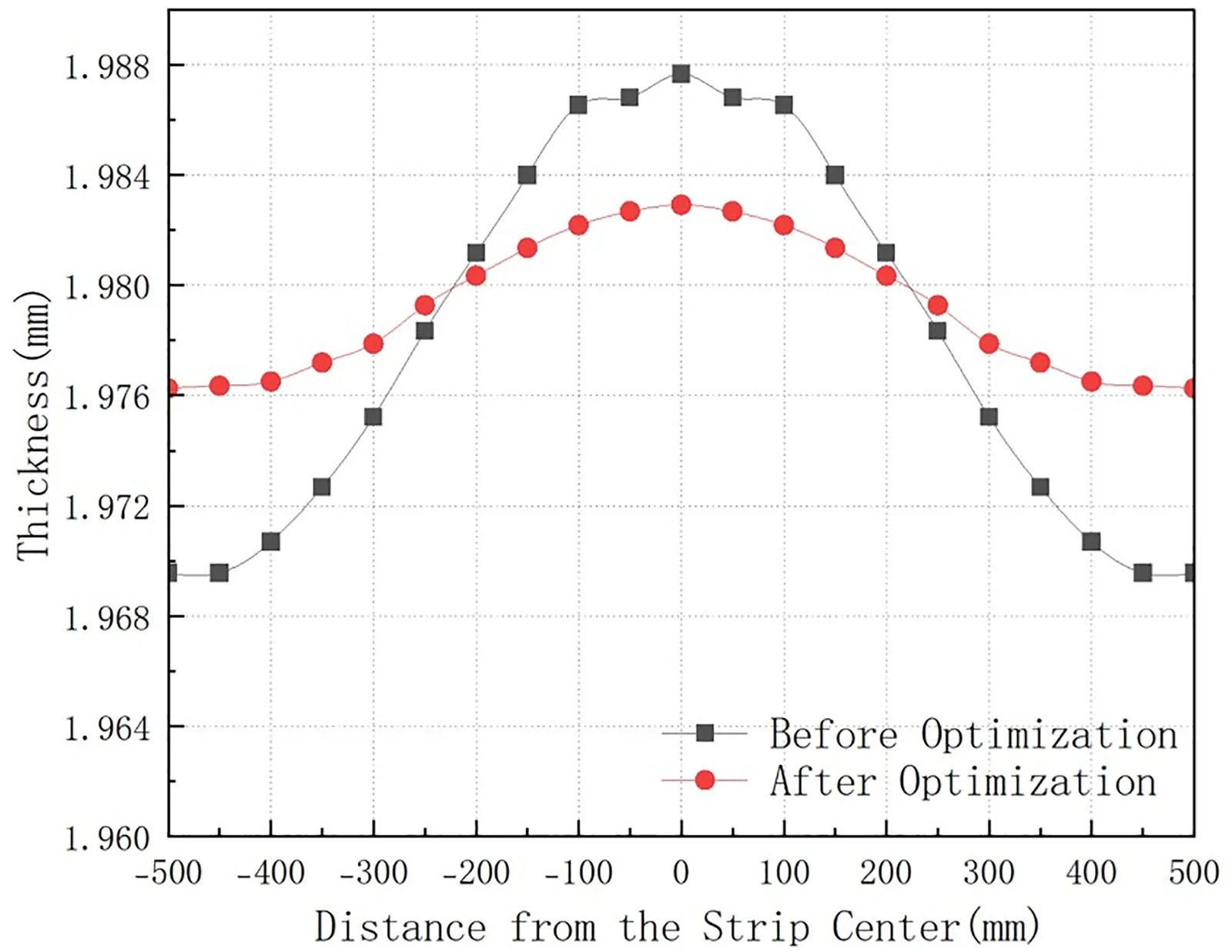
Comparison of thickness optimization effects.

As can be visually observed from Fig 23, the thickness distribution before optimization exhibits a distinct feature of being thinner in the center and thicker at the edges. The center thickness is 1.988 mm, while the edge thickness reaches 1.97 mm, with a thickness difference of 0.018 mm. This uneven thickness distribution is primarily attributed to the excessive bending deformation of the work roll under the influence of high rolling force, leading to instability in the shape of the roll gap. Under heavy-load conditions, the rolling force causes excessive bending deformation of the work roll, resulting in an unstable roll gap shape that is concave. The reduction in the central region of the strip steel is significantly less than that at the edges, ultimately forming a center-thick, edge-thin, concave thickness distribution. This is highly prone to causing severe mid-wave defects, seriously affecting the shape quality and performance of the strip steel.

The optimized thickness distribution curve tends to be flatter, with the center thickness reaching 1.982 mm and the edge thickness reaching 1.976 mm, and the thickness variation is reduced to 0.006 mm. The optimized process parameters effectively balance the roll deformation caused by the large rolling force, making the roll gap shape tend to be straight, and the reduction within the full width of the strip steel is uniform. This fundamentally eliminates the concave thickness distribution, laying a solid foundation for excellent plate shape quality.

By coordinating the bending forces of the work roll and the intermediate roll, the counter-bending moment generated by the positive bending force is utilized to precisely offset the excessive bending deformation of the work roll caused by the large rolling force. At the same time, the high stiffness regulation characteristics of the intermediate roll are matched, so that the roll gap shape is restored from an unstable concave shape to a flat state, ultimately achieving uniform distribution of thickness across the full width of the strip steel.

#### 5.2.2 Crown optimization effect.

Crown control is the core of shape quality, directly affecting the cross-sectional shape of the strip and subsequent processing performance. Optimized crown control achieves fine control from single crown to composite crown.

**Fig 24:**
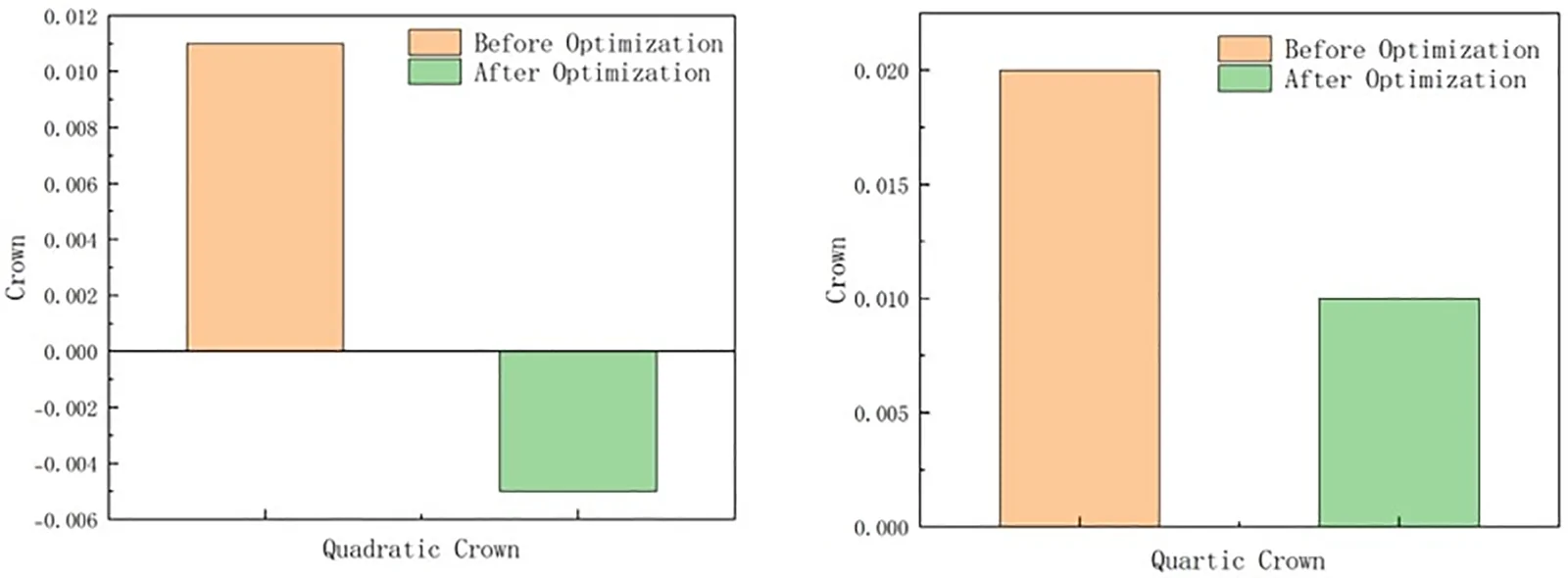
Comparison of crown optimization effects.

As can be intuitively observed from Fig 24, the secondary crown has been improved from +11μm before optimization to −5μm after optimization, which is closer to the ideal state of zero crown. By coordinating the optimization of the work roll and intermediate roll bending forces, the excessive bending of the roll system caused by large rolling forces has been effectively offset, restoring the roll gap from a convex shape to a nearly flat state. This fundamentally corrects the macro thickness distribution of the strip steel and eliminates macro shape defects. The fourth-order crown has been optimized from 3μm to 1μm, achieving a breakthrough improvement. On the basis of correcting the macro secondary crown, by matching the high stiffness and gradual adjustment characteristics of the intermediate roll bending force, the thickness deviation caused by inter-roll flattening, local deformation, etc. has been precisely compensated, eliminating local thickness gradients and achieving fine flatness control across the full width of the strip steel.

#### 5.2.3 Flatness optimization effect.

Flatness is the comprehensive embodiment of shape quality, directly affecting product performance and appearance quality. Optimized flatness control achieves a transformation from macro-level flatness to micro-level precision.

**Fig 25:**
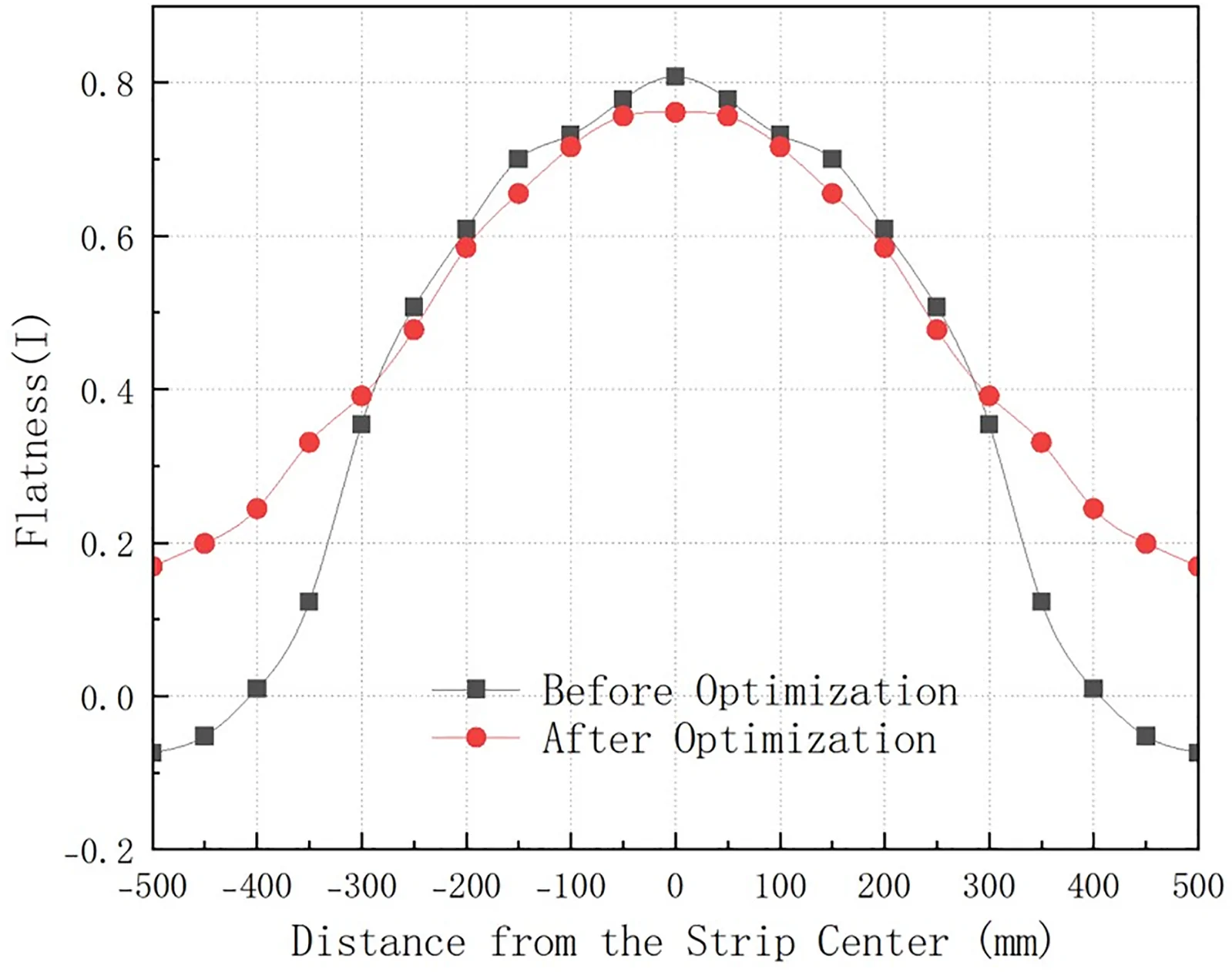
Comparison of flatness optimization effects.

As can be seen from Fig 25, the flatness distribution before optimization exhibits obvious residual wave patterns, primarily manifesting as slight edge wave characteristics. The thickness reduction and elongation rate at the edge of the strip steel are greater than those in the central area, leading to residual compressive stress at the edges and ultimately forming edge wave defects, which seriously affect the plate shape quality and performance of the strip steel. The optimized flatness distribution is basically close to the zero position line. The optimized process parameters effectively balance the elongation rate and stress distribution across the full width of the strip steel, fundamentally eliminating edge wave defects and providing direct guarantee for the excellent plate shape of the strip steel. Through process optimization, the shape of the roll gap is precisely controlled, making the reduction across the full width of the strip steel more consistent, eliminating the difference in elongation rate between the edges and the central area, and thus eliminating residual compressive stress. This fundamentally solves the edge wave defects.

#### 5.2.4 Rolling force distribution optimization effect.

The uniformity of rolling force distribution not only affects shape quality but also directly relates to equipment life and production safety. Optimized load distribution achieves a win-win for both quality and equipment.

**Fig 26:**
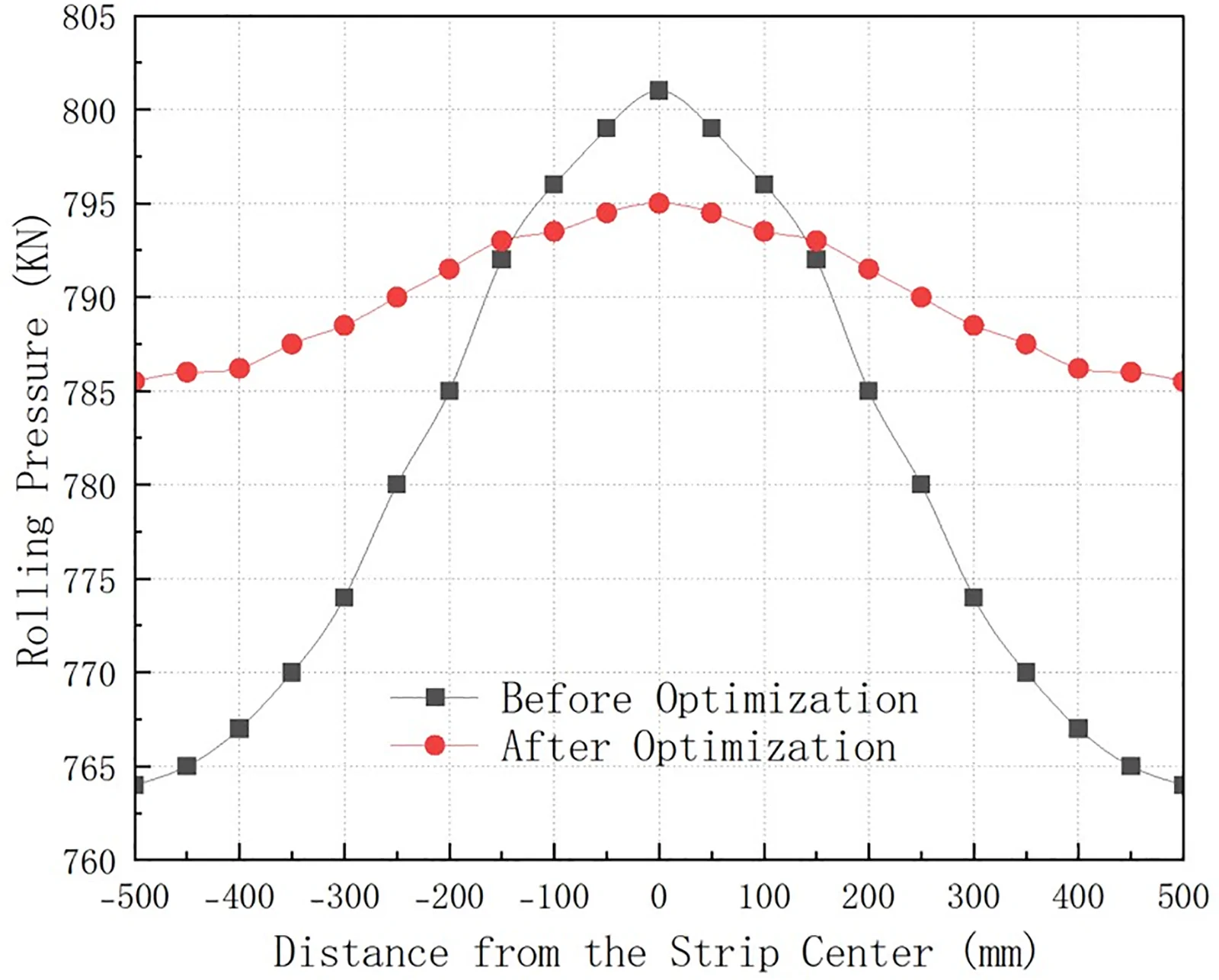
Comparison of rolling force distribution optimization effects.

As can be seen from Fig 26, the distribution before optimization exhibits a pronounced edge concentration phenomenon, with the edge rolling force reaching 788 MPa and the central area at 810 MPa, resulting in a non-uniformity of 8.7%. Under high rolling forces, excessive bending of the roll system leads to increased roll gap crown, insufficient reduction in the central area, concentrated load, and excessive reduction and low load at the edges, ultimately forming an extremely uneven rolling pressure distribution. This further exacerbates the imbalance in roll system deformation, creating a vicious cycle of “deformation – load.” The optimized distribution tends to be flatter, with the edge rolling force reduced to 785 MPa and the central area at 795 MPa, and the non-uniformity decreased to 2.1%, representing an improvement of 75.9%. The optimized process parameters effectively reconstruct the force balance of the roll system. By precisely controlling the roll gap shape, the reduction across the full width of the strip steel tends to be consistent, fundamentally eliminating the problems of overload in the central area and insufficient load at the edges. This breaks the vicious cycle of “deformation – load” and provides a guarantee for the stable operation of the roll system. Through coordinated bending force control, the process optimization precisely counteracts the excessive bending of the work roll caused by high rolling forces, restoring the roll gap from a convex shape to a flat state. The reduction across the full width of the strip steel tends to be consistent, eliminating the lateral differences in rolling pressure from the root cause. The uniform rolling pressure further suppresses the additional deformation of the roll system, forming a positive cycle of “flat roll gap → uniform reduction → uniform load → more stable roll gap,” continuously improving the stability of the rolling process.

The globally optimal collaborative bending force combination solved by the NSGA-II multi-objective optimization is: WRB = 125 kN, IRB = 160 kN. This set of coordinated bending forces achieves the comprehensive optimal balance of thickness uniformity, crown, flatness and rolling force distribution under the 25000 kN high rolling force constraint.

## 6. Conclusion

Through theoretical analysis and finite element simulation, this paper systematically studied the shape generation mechanisms and control strategies of a six-high cold tandem rolling mill under high rolling force conditions, reaching the following main conclusions:

1. Ultra-high rolling force intensifies elastic bending and flattening deformation of work rolls and intermediate rolls, causing obvious deviation of roll gap profile and shifting the optimal single WRB/IRB operating window; the proportion of quartic crown rises significantly, and traditional single bending force regulation cannot fully compensate roll system deformation.
2. Intermediate roll bending force possesses stronger anti-disturbance ability under heavy load, with outstanding regulation effect on quarter-wave defects and quartic crown; collaborative coordination of WRB and IRB is a necessary condition to realize full-width uniform strip shape under extreme rolling force.
3. The proposed LHS-GPR/RBF surrogate model coupled NSGA-II-PSO multi-objective optimization strategy realizes synchronous optimization of dual bending forces. After optimization, transverse thickness difference, quadratic/quartic crown residual value, flatness wave amplitude and rolling pressure unevenness are greatly improved, which verifies the engineering practicability of the method and provides a parameter optimization framework for six-high cold rolling mills under high rolling force.

## Supporting information

S1 File Supplementary Data. (ZIP)
